# Priming with a Seaweed Extract Strongly Improves Drought Tolerance in Arabidopsis

**DOI:** 10.3390/ijms22031469

**Published:** 2021-02-02

**Authors:** Fiaz Rasul, Saurabh Gupta, Justyna Jadwiga Olas, Tsanko Gechev, Neerakkal Sujeeth, Bernd Mueller-Roeber

**Affiliations:** 1 Institute of Biochemistry and Biology, University of Potsdam, Karl Liebknecht Str. 24-25, 14476 Potsdam-Golm, Germany; rasul@uni-potsdam.de (F.R.); gupta@mpimp-golm.mpg.de (S.G.); olas@uni-potsdam.de (J.J.O.); 2 BioAtlantis Ltd., Clash Industrial Estate, V92 RWV5 Tralee, Ireland; 3 Max Planck Institute of Molecular Plant Physiology, Am Mühlenberg 1, 14476 Potsdam-Golm, Germany; 4 Center of Plant Systems Biology and Biotechnology (CPSBB), 139 Ruski Blvd., 4000 Plovdiv, Bulgaria; gechev@cpsbb.eu; 5 Department of Plant Physiology and Molecular Biology, University of Plovdiv, 24 Tsar Assen Str., 4000 Plovdiv, Bulgaria

**Keywords:** abiotic stress, *Ascophyllum nodosum*, drought, priming, reactive oxygen species

## Abstract

Drought represents a major threat to plants in natural ecosystems and agricultural settings. The biostimulant Super Fifty (SF), produced from the brown alga *Ascophyllum nodosum*, enables ecologically friendly stress mitigation. We investigated the physiological and whole-genome transcriptome responses of *Arabidopsis thaliana* to drought stress after a treatment with SF. SF strongly decreased drought-induced damage. Accumulation of reactive oxygen species (ROS), which typically stifle plant growth during drought, was reduced in SF-primed plants. Relative water content remained high in SF-treated plants, whilst ion leakage, a measure of cell damage, was reduced compared to controls. Plant growth requires a functional shoot apical meristem (SAM). Expression of a stress-responsive negative growth regulator, *RESPONSIVE TO DESICCATION 26* (*RD26*), was repressed by SF treatment at the SAM, consistent with the model that SF priming maintains the function of the SAM during drought stress. Accordingly, expression of the cell cycle marker gene *HISTONE H4* (*HIS4*) was maintained at the SAMs of SF-primed plants, revealing active cell cycle progression after SF priming during drought. In accordance with this, *CYCP2;1*, which promotes meristem cell division, was repressed by drought but enhanced by SF. SF also positively affected stomatal behavior to support the tolerance to drought stress. Collectively, our data show that SF priming mitigates multiple cellular processes that otherwise impair plant growth under drought stress, thereby providing a knowledge basis for future research on crops.

## 1. Introduction

Climate change represents a major threat to food security and can negatively affect crop yields [1,2]. Environmental factors such as drought and heat significantly reduce terrestrial net primary production [3,4,5,6]. In the past decade, global losses in crop production due to drought totaled approximately USD 29 billion between 2005 and 2015 [7]. A combination of bioengineering [8] and other sustainable treatment technologies is required to boost agricultural yields despite increased drought and water scarcity. Treatment technologies using “biostimulants” may potentially fine-tune drought response pathways while preserving yield in agriculture [9].

Extracts produced from marine algae, particularly those derived from the cold-water seaweed *Ascophyllum nodosum* (family Fucaceae), are generally regarded as biostimulants. According to the European Biostimulants Industry Council (EBIC), a biostimulant, when applied to plants, seeds, soil, or growth media, improves the response to stress, provides benefits to plant development, and improves nutrient-use efficiency (EBIC, www.biostimulants.eu) [9,10]. Biostimulants, used as a sustainable means to improve yield and the plant’s performance under environmental stress conditions, have received considerable interest in recent years [10,11,12,13].

Seaweed extract under the trade name Super Fifty (SF) is a concentrated alkaline extract of *A. nodosum* (500 g/L) [14]. *A. nodosum* extracts are rich in polyphenols and unique polymers such as fucoidan and alginates [15,16], some of which are known for their antioxidant activities which may directly quench otherwise harmful reactive oxygen species (ROS) [14,17,18,19]. Although it is well-known that *A. nodosum* extracts are active on higher plants and can support their growth and resilience to abiotic stresses, knowledge about the underlying molecular mechanisms is currently largely missing [12,14,20,21,22].

Molecular priming is an approach to induce inherent plant defense systems by various organic or inorganic molecules and biostimulants, resulting in protection against subsequent stresses [22]. Here, we report that *Arabidopsis thaliana* plants primed with SF exhibit high tolerance to drought stress, retain high relative water content (RWC) in leaves with reduced ROS accumulation, and also exhibit reduced membrane damage compared to unprimed drought-stressed plants. By performing a global transcriptome analysis using RNA-seq, we identified possible mechanisms underlying the SF-triggered protection against drought stress. In accordance with the protective function of SF against water limitation, we found that drought-specific signaling networks are affected in SF-primed plants to activate stress tolerance mechanisms.

Plant vegetative growth and transition to reproductive growth requires a functional shoot apical meristem (SAM) [23]. Considering this, we found that expression of NAC transcription factor *RESPONSIVE TO DESICCATION 26* (*RD26*), a stress-responsive negative growth regulator [24,25], is strongly induced in the SAMs of drought-stressed plants, while interestingly this induction is almost completely mitigated by SF priming. In addition, expression of the cell cycle marker gene *HISTONE H4* (*HIS4*) at the SAM is strongly repressed during drought, but this effect is fully reversed by SF priming. The survival under drought also requires a timely closure of stomata in the leaf epidermis. In accordance with this, priming with SF triggers a timely reduction in stomatal aperture, helping to maintain tissue water level under drought. Collectively, our data provide insights into the molecular responses affected by SF priming and helping plants to withstand drought stress.

## 2. Results

### 2.1. SF Priming Enables Arabidopsis to Overcome the Effects of Drought

**Figure 1:**
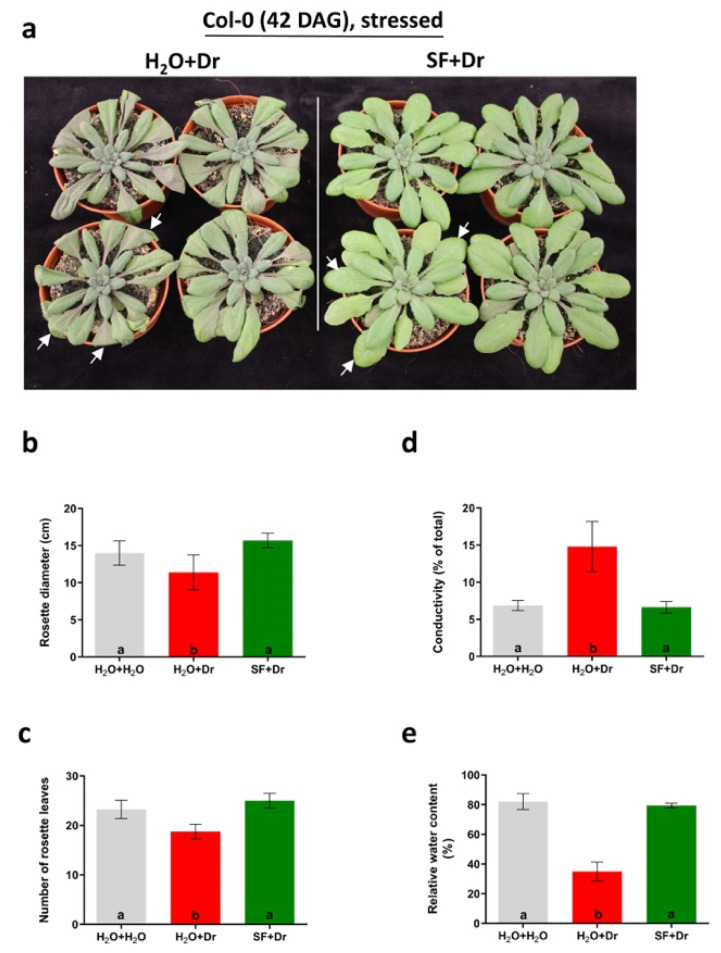
Response of Super Fifty (SF)-primed and unprimed plants to drought stress. (**a**) Arabidopsis plants after 11 days of drought stress, at 42 days after germination (DAG). In the absence of SF priming, drought-stressed plants (H_2_O+drought stress (Dr)) show stress-related symptoms (wilting of most leaves), while drought stress-related symptoms are seemingly absent in SF-primed plants (SF+Dr). Leaves no. 8, 9, and 10, harvested for ion conductivity and relative water content (RWC) measurements—see panels (**d**,**e**)—are indicated by white arrows (representative images). (**b**) Rosette diameter. (**c**) Number of rosette leaves. Data in (**b**,**c**) were obtained for plants at 40 DAG. (**d**) Ion conductivity in leaves as a measure of membrane damage. (**e**) Relative water content in leaves. Error bars denote the standard deviation of the mean (SD). Treatments sharing same letters indicate nonsignificant differences, while treatments with different letters indicate significant differences (*p* < 0.05; one-way ANOVA, Tukey’s multiple comparison test). Dr, drought stress; SF, Super Fifty.

To determine whether pretreatment of plants with SF as a priming agent can induce tolerance against drought stress, a drought experiment was performed using Arabidopsis as a model, following the protocol shown in Supplementary Figure S1. Arabidopsis Col-0 plants were primed twice with SF at a concentration of 0.2% (v/v) each. The first foliar spray was applied 25 days after germination (DAG), followed by a second spray at 27 DAG. From 31 to 42 DAG, plants were subjected to drought stress. Plants primed in this way showed better drought tolerance than control plants not pretreated with SF. Control plants started wilting after 8 days of continuous drought, and symptoms were more pronounced after 11 days of stress (Figure 1a). In contrast, SF-primed plants showed improved tolerance to drought (Figure 1a). Significant differences in development were observed, including a bigger rosette diameter and a higher number of rosette leaves in SF-primed than unprimed plants (Figure 1b,c). We also determined electrolyte leakage (EL) as a measure of cell membrane damage due to drought stress and RWC at day 11 after drought establishment. EL of primed plants was significantly lower (*p* < 0.05) than that of unprimed plants exposed to drought (Figure 1d). A severe reduction in RWC was observed in unprimed plants after 11 days of continuous drought, which was mitigated by SF priming (Figure 1e). The results demonstrate a strongly improved tolerance to drought stress in plants primed with SF.

### 2.2. SF Priming Lowers Hydrogen Peroxide Levels in Plants

**Figure 2:**
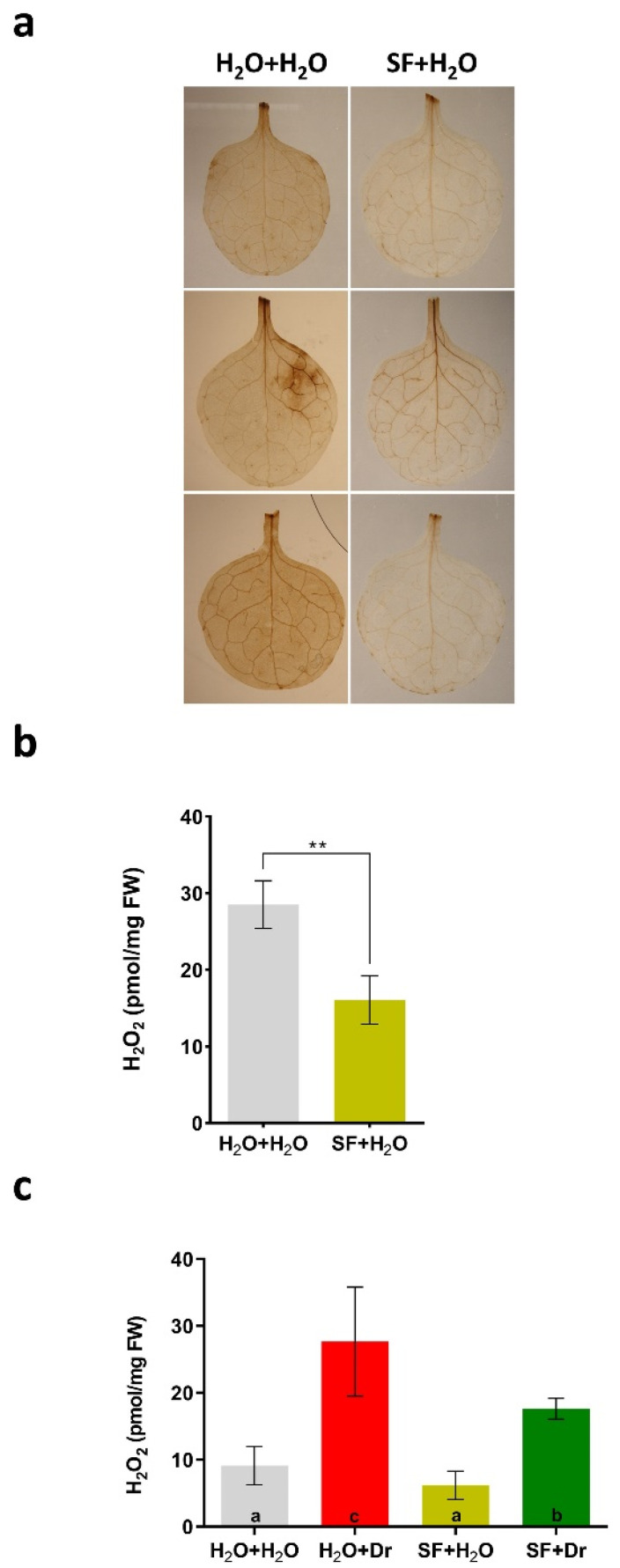
SF priming triggers a reduction in reactive oxygen species (ROS) levels. (**a**) Histochemical staining of ROS in leaves using 3,3′-diaminobenzidine (DAB). (**b**) Hydrogen peroxide levels in leaves of 15-day-old Arabidopsis Col-0 plants without (H_2_O+H_2_O) and with SF treatment (SF+H_2_O) in the absence of drought stress. (**c**) Hydrogen peroxide levels in leaves at 42 DAG. Note the higher H_2_O_2_ levels in unprimed plants (H_2_O+Dr) compared to SF-primed plants (SF+Dr) under drought stress. Quantification of H_2_O_2_ levels (**b**,**c**) was performed using the Amplex Red Hydrogen Peroxide/Peroxidase kit as reported [30]. Error bars in (**b**,**c**) denote the standard deviation of the mean (SD). In (**b**), asterisks (**) indicate significant difference (*n* = 4) (*p* < 0.01, Student’s *t*-test). In (**c**), different letters indicate statistically significant differences between the samples (*p* < 0.05, *n* = 8; one-way ANOVA, Tukey’s multiple comparison test). FW, fresh weight; Dr, drought stress; SF, Super Fifty.

Hydrogen peroxide (H_2_O_2_) is an important stress molecule that accumulates to often toxic levels in many abiotic stresses including drought [26,27], while moderate levels of H_2_O_2_ have a signaling function in plants [28,29]. Avoiding high levels of H_2_O_2_ is, thus, important to keep plants healthy and for maintaining growth. Having observed the beneficial effect of SF on drought-stressed plants, we considered that SF priming might affect H_2_O_2_ levels. Therefore, H_2_O_2_ levels were determined by 3,3’-diaminobenzidine (DAB) staining in young Arabidopsis leaves (first leaf pair) from 15-day-old plants. In the absence of drought, SF-treated leaves showed weaker DAB staining than non-SF-treated plants, indicating lower H_2_O_2_ accumulation (Figure 2a). This observation was confirmed by Amplex Red assay (Figure 2b). Furthermore, H_2_O_2_ levels were determined in rosettes of Arabidopsis plants subjected to drought stress in the absence or presence of SF treatment. While in the absence of SF priming H_2_O_2_ levels strongly increased in drought-stressed plants, this increase was significantly reduced in SF-treated plants (Figure 2c). We concluded that SF priming improves tolerance to drought stress by preventing H_2_O_2_ accumulation.

### 2.3. SF Priming Alters Global Gene Expression Patterns During Drought Stress

To understand how SF minimizes the effect of drought stress, we performed transcriptome profiling using plants which were drought-stressed for 11 days (42 DAG) in the absence or presence of SF priming. Control plants were kept well-watered (see above; Supplementary Figure S1). Leaf samples were collected and subjected to RNA-seq.

**Figure 3:**
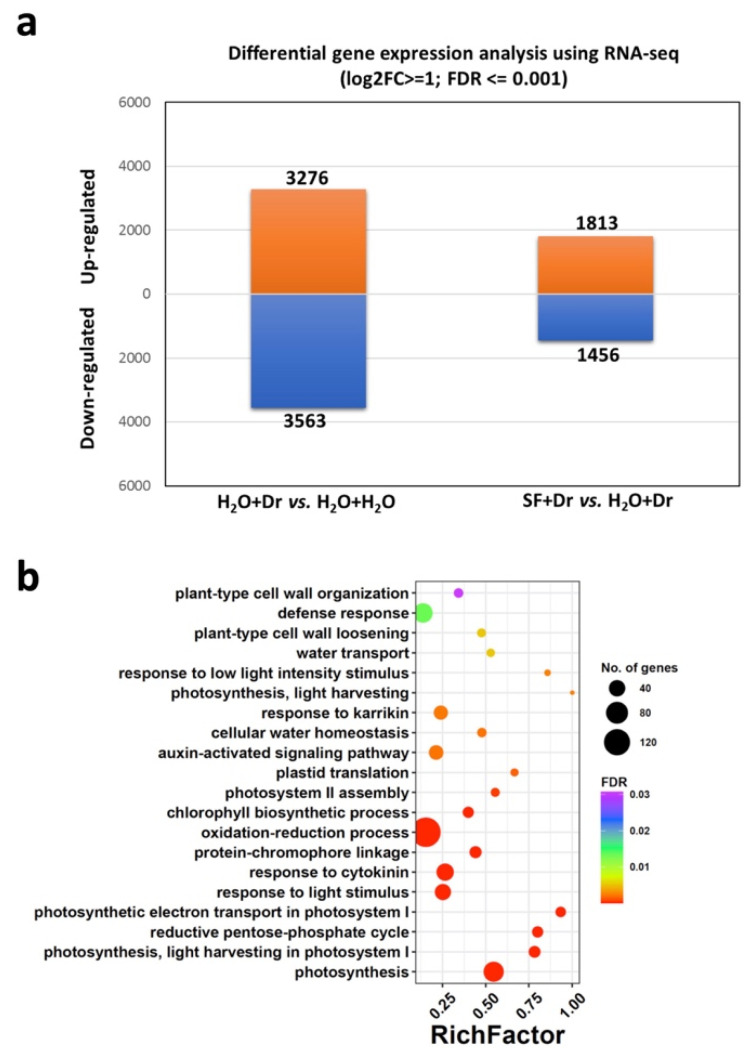
SF priming-induced transcript changes during drought stress in Arabidopsis. (**a**) Number of differentially expressed genes (DEGs) in stressed (H_2_O+Dr) vs. unstressed (H_2_O+H_2_O), and in primed (SF+Dr) vs. unprimed (H_2_O+Dr) plants during drought. The list of DEGs is provided in Supplementary File S1. (**b**) Gene ontology (GO) enrichment of DEGs in SF-primed and stressed (SF+Dr) plants categorized into different groups based on the biological process. The RichFactor represents the ratio of the number of DEGs annotated with the given GO term to the number of all genes annotated with the respective GO term. The sizes and colours of the dots represent the number of genes and FDR value, respectively. The list of enriched GO terms is provided in Supplementary File S2. Dr, drought stress; SF, Super Fifty.

To determine the transcriptional changes induced by drought and SF treatment, a pairwise differential expression analysis was performed considering all treatment combinations (Supplementary File S1). In the absence of SF priming, many genes (6839 genes in total: 3276 up- and 3563 downregulated) were differentially expressed upon drought stress (H_2_O+ drought stress (Dr) vs. H_2_O+H_2_O; Figure 3a), in accordance with published data [31]. Fewer genes (2259 in total: 1261 up- and 998 downregulated; Supplementary File S1) were affected by drought stress after SF priming (SF+Dr vs. H_2_O+H_2_O), in accordance with the growth phenotype observed (Figure 1). Furthermore, upon drought stress, the SF-primed plants (SF+Dr) exhibited a large number of differentially expressed genes (3269 genes in total: 1813 up- and 1456 downregulated; Figure 3a) as compared to unprimed plants (H_2_O+Dr). In the absence of drought stress, SF affected the expression of only 195 genes (SF+H_2_O vs. H_2_O+H_2_O), of which the vast majority (193 genes) were upregulated (Supplementary File S1).

Genes showing a significant change in expression in the pairwise differential expression analysis (Supplementary File S1) were further analyzed using the gene ontology (GO) enrichment tool GOSeq (Supplementary File S2). The GO terms “response to water deprivation” (GO:0009414), “response of abscisic acid (ABA)” (GO:0009737), and “oxidation-reduction process” (GO:0055114) were notably enriched in terms of biological processes in SF-primed and unstressed (SF+H_2_O) vs. unprimed and unstressed (H_2_O+H_2_O) plants suggesting that priming-related genes are induced by SF (Supplementary File S2). Biological processes related to “response to oxidative stress” (GO:0006979), “plant-type cell wall loosening” (GO:0009828), “leaf senescence” (GO:0010150), and “response to abscisic acid (ABA)” (GO:0009737) were enriched in SF-primed and drought-stressed plants (SF+Dr) vs. unprimed and drought-stressed plants (H_2_O+Dr) (Supplementary File S2). When SF-primed and drought-stressed plants (SF+Dr), which showed drought tolerance, were compared to unprimed and unstressed plants (H_2_O+H_2_O), an enrichment of the GO terms “response to water deprivation” (GO:0009414), “response to salt stress” (GO:0009651), “oxidation-reduction process” (GO:0055114), “cell wall biogenesis” (GO:0042546), and “response to gibberellin” (GO:0009739) was observed (Supplementary File S2). Drought stress on SF-primed plants (SF+Dr vs. SF+H_2_O) significantly induced genes related to “regulation of transcription, DNA-templated” (GO:0006355), “response to ABA” (GO:0009737), “response to water deprivation” (GO:0009414), “response to salt stress” (GO:0009651), and “response to cold” (GO:0009409). Furthermore, genes related to “plant-type cell wall loosening” (GO:0009828), “oxidation-reduction process” (GO:0055114), “chlorophyll biosynthetic process” (GO:0015995), “water transport” (GO:0006833), and “defense response” (GO:0006952) were upregulated in SF-primed (SF+Dr) vs. unprimed drought-stressed plants (H_2_O+Dr) (Figure 3b). This elevation of key biological processes related to the response to water deprivation and the defense to ROS in SF-primed and drought-stressed plants might explain the improved drought tolerance of SF-primed plants. Based on the enrichment analysis, we then selected differentially expressed genes (DEGs) associated with drought and hormone signaling pathways, ROS metabolism, and stomatal defense (Table 1).

With respect to priming, an interesting question regards which genes are affected by SF treatment already in the absence of drought stress (i.e., in the comparison SF+H_2_O vs. H_2_O+H_2_O). These genes include the stress-related genes *GLUTATHIONE S-TRANSFERASE TAU 4* (*GSTU4*), *SENESCENCE-ASSOCIATED GENE 13* (*SAG13*), *RAFFINOSE SYNTHASE 5* (*RFS5*), several late embryogenesis abundant (LEA) protein-coding genes (*AT1G52690*, *AT2G42560*, AT3G02480, and AT5G06760), and genes coding for membrane protein aquaporins, *PIP2-3* and *PIP2B*, which are involved in the transport of water and other solutes across membranes, as well as lipid-transfer proteins AZI3 and EARLI1 (Table 1). Although the GO term categories (see above) report an overrepresentation of “response to ABA” (GO:0009737), several additional genes link the biostimulant SF to other plant hormones as well. For example, the SF-mediated induction of *NITRILASE 2* (*NIT2*), which is involved in auxin biosynthesis [32], indicates a possible involvement of auxin signaling in the priming process. Of note, most of the above genes are much less induced during drought stress in SF-primed (SF+Dr) than nonprimed plants. An exception are the water and lipid-transfer protein genes *PIP2-3*, *PIP2B*, *AZI3*, and *EARLI1*, which all showed higher expression (4- and 13-fold) in SF-primed, drought stress plants (SF+Dr) than in plants subjected to drought stress in the absence of SF priming (H_2_O+Dr). This observation indicates an important role of the metabolite transport genes for establishing the superior drought tolerance observed in plants after SF priming.

### 2.4. SF Priming Induces ABA-Dependent Drought Signaling

Phytohormones, signaling proteins, and transcription factors (TFs) act together to form a web of interactions at the molecular level during drought. The drought-affected signaling pathways in plants are broadly classified into two categories—ABA-dependent and ABA-independent [33,34,35,36]. The RNA-seq data revealed changes of genes encoding TFs and proteins linked to drought signaling in SF-primed plants. Genes related to “response to abscisic acid” (GO:0009737) were downregulated in SF+Dr compared to H_2_O+Dr (Supplementary File S2). The expressions of genes encoding a protein phosphatase 2C family protein (PP2C52), a protein kinase superfamily protein (SnRK2.8), and an abscisic acid-responsive element-binding factor (ABF3) were altered due to SF priming (Table 1). All these regulatory components are linked to ABA-dependent drought signaling (Supplementary Figure S2). Under drought, *PP2C52* was strongly downregulated in unprimed plants, while its expression was much less affected (i.e., reduced) by drought in plants previously primed with SF (Table 1). The PP2C52 protein interacts with the β-subunit of a heterotrimeric GTP-binding protein (AGB1) in the plasma membrane and transmits signals via dephosphorylation of other proteins [37]. Notably, AGB1 is a positive regulator of drought tolerance and activates ROS detoxification [38]. On the contrary, genes encoding plant PP2Cs belonging to the group A subfamily, such as *ABI1* and *ABI2*, and the ABA-induced PP2C gene *HAI3*, were strongly upregulated during drought stress, while their expressions were considerably less induced in SF-primed plants under drought (Table 1). Both, ABI1 and ABI2 are known negative regulators of the ABA response in Arabidopsis [39]. Similarly, HAI3 is a negative regulator of osmoregulatory solute accumulation and drought resistance. The knockout mutant of *HAI3* had increased proline and osmoregulatory solute accumulation when soil water potential decreased during drought [40]. Thus, the effect of SF on the expression of the ABA signaling pathway genes strongly indicates an activation of core ABA signaling for drought tolerance in SF-primed and drought-stressed plants.

Another classical regulator of ABA signaling downstream of PP2C is SNF1-related protein kinase 2 (subclass III SnRK2) [41]. In the absence of SF priming, the gene encoding SnRK2.8 was downregulated 2.7-fold in drought-stressed plants compared to well-watered plants, while in the presence of SF priming its expression was 8.5-fold higher during drought compared to nonprimed plants (Table 1). In addition, the gene encoding its downstream interacting partner ABF3 was upregulated ~2-fold in SF-primed plants (vs. unprimed plants), both in the absence and presence of drought stress (Table 1). The elevated expression of *SnRK2.8* after SF priming and the superior drought tolerance of SF-primed plants are well in accordance with the observation that overexpression of *SnRK2.8* leads to drought tolerance in Arabidopsis [42]. SnRK2.8 also plays a role in metabolic processes related to plant growth [43]. SnRK2.8 is a nuclear-localized protein and a regulatory component of ABA-dependent signaling. Upon exposure to drought, it phosphorylates ABA-responsive element-binding factors (ABFs) which leads to an ABA-dependent activation of stress-responsive genes [44,45].

Genes coding for dehydration-responsive element binding proteins, DREB1A and DREB3 (TINY2), were activated in SF-primed plants exposed to drought (Table 1). DREB proteins function in the ABA-dependent pathway [46]. Transgenic *Salvia miltiorrhiza* (Chinese Sage) plants expressing *DREB1A* from Arabidopsis gained tolerance to drought [47]. DREB3 belongs to the DREB subfamily A-4 of the AP2/ERF transcription factor family and its expression is induced by drought and salt stress [48]. We did not observe induction of *DREB3* in unprimed drought-stressed plants while it was induced in SF-primed drought-stressed plants compared to stressed and nonstressed unprimed plants, suggesting a role in SF priming-induced drought tolerance (Table 1).

Genes coding for nuclear factor Y (NF-Y) TFs were also upregulated in SF-primed plants exposed to drought, specifically *NF-YB2, NF-YB3,* and *NF-YA10* (Table 1). NF-Y proteins take part in the drought stress response in an ABA-dependent manner and activate drought responsiveness by binding the CCAAT box in target gene promoters [36]. Transgenic maize plants with increased *ZmNF-YB2* expression showed drought tolerance, increased chlorophyll content, and improved grain yield [49]. In Arabidopsis, the knockout mutant *nf-yb3* is sensitive to drought and exhibits lower water-use efficiency [50]. The NF-YA10 TF has a role in leaf growth, and Arabidopsis *35S-NF-YA10* overexpressors showed enhanced biomass accumulation and increased cell expansion compared to controls [51]. Taken together, the selective changes in the expression of genes encoding PP2C, SnRK, ABF, DREB, and NF-Y family members strongly suggest activation of ABA-dependent drought signaling in SF-primed plants.

### 2.5. SF Priming Triggers Expression of Transcription Factor Gene ERF53

Transcription factor ERF53 belongs to the APETALA2/ETHYLENE RESPONSIVE FACTOR (AP2/ERF) superfamily which plays a role in stress responses and plant development [52]. The RING domain of ubiquitin E3 ligases RGLG1 and RGLG2 target ERF53 for proteasomal degradation [53]. Overexpression of *ERF53* in the *rglg1 rglg2* double knockout mutant improves drought tolerance [53]. Furthermore, ABA levels are elevated in *ERF53* overexpression lines compared to *rglg1 rglg2* [54]. Interestingly, in both stressed and nonstressed SF-primed plants, *ERF53* was upregulated while *RGLG1* and *RGLG2* were downregulated compared to drought-stressed plants (Table 1), suggesting that priming with SF triggers drought stress tolerance by modulating the ERF53 regulatory network along with an activation of ABA-dependent drought signaling. Notably, we also observed a priming-specific upregulation of other *ERF* genes related to stress responses and plant development (*ERF2, ERF54,* and *ERF94*) [52] in SF-treated plants (Table 1). The specific roles these ERFs play in SF priming-induced responses in Arabidopsis requires further investigation.

### 2.6. SF Priming Affects Expression of ROS-Related Genes

Given that SF priming reduces the accumulation of ROS (Figure 2), we investigated the expression of 180 H_2_O_2_-, superoxide (O_2_^•−^)-, and singlet oxygen (^1^O_2_)-responsive marker genes [55] and observed differential expressions of 118 of them (Supplementary File S3); 72 genes were significantly downregulated in SF-primed and drought-stressed plants (SF+Dr) compared to plants subjected to drought in the absence of SF priming (H_2_O+Dr) (Supplementary File S3). This observation confirms that hallmark oxidative stress genes are predominantly repressed by SF in plants exposed to drought. Moreover, of 217 genes linked to antioxidant- and ROS-related enzymes [55], 75 genes were differentially expressed, of which 21 were upregulated in SF-primed plants exposed to drought (SF+Dr) compared to only drought-stressed plants (H_2_O+Dr; Supplementary File S3). The differentially expressed genes code for proteins involved in regulating redox state and cellular ROS homeostasis.

The AGC protein kinase gene *OXIDATIVE SIGNAL-INDUCIBLE1* (*OXI1*) is involved in plant responses to oxidative signals and is induced by ROS [56,57]. Interestingly, expression of *OXI1* and another oxidative burst-associated gene, *RESPIRATORY BURST OXIDASE HOMOLOG C* (*RBOHC*, also called *RHD2*), was highly upregulated by drought, a response mitigated by prior SF treatment (Table 1).

The ascorbate-glutathione cycle is one of the major antioxidant systems for ROS detoxification in plant cells [58,59]. In this cycle, ascorbate peroxidase (APX) converts H_2_O_2_ to water using reduced ascorbate (ascorbic acid, AsA) as an electron donor, resulting in monodehydroascorbate (MDHA) and dehydroascorbate (DHA), which are then recycled back to AsA using reduced glutathione (GSH) and catalyzed by dehydroascorbate reductase (DHAR). Finally, the oxidized glutathione (GSSG) is reduced back to GSH by glutathione reductase. Here, we observed a considerably increased expression (3.6- to 7.7-fold) of chloroplastic ascorbate peroxidases (*APX4* and *TAPX*) and *DHAR3* in droughted SF-primed plants (SF+Dr) vs. nonprimed plants (H_2_O+Dr) (Table 1). DHAR3 is involved in ascorbate-glutathione cycle-mediated redox regulation and ROS scavenging in chloroplasts [60]. Although APX4 lacks obvious catalytic and heme-binding domains, soluble APX activity is reduced and H_2_O_2_ level is elevated in *apx4* null mutants, indicating a role in ROS scavenging [61]. Interestingly, recent studies have shown that thylakoid ascorbate peroxidase (tAPX) functions as a regulatory hub that memorizes a cold priming stimulus, controls ROS signaling, and improves plant performance upon future stress [62,63]. The *TAPX* gene was expressed at higher levels in SF-primed and drought-stressed plants than in only drought-stressed plants. Additionally, a gene encoding glutathione peroxidase 1 (GPX1) was upregulated in SF-primed plants. GPX1 is mainly involved in the glutathione peroxidase cycle whereby H_2_O_2_ is detoxified to H_2_O, driven by the oxidation of ascorbate to MDHA [64]. Additionally, a glutathione transferase encoding gene (*GSTL2*; Table 1), belonging to the lambada class of the glutathione transferase family and predicted to have a role in redox homeostasis [65], was selectively upregulated in SF-primed plants. The upregulated expression of *APX4, TAPX, DHAR3, GPX1,* and *GSTL2* likely reduces the effect of oxidative stress in SF-primed plants and, thereby, improves their drought tolerance.

**Figure 4:**
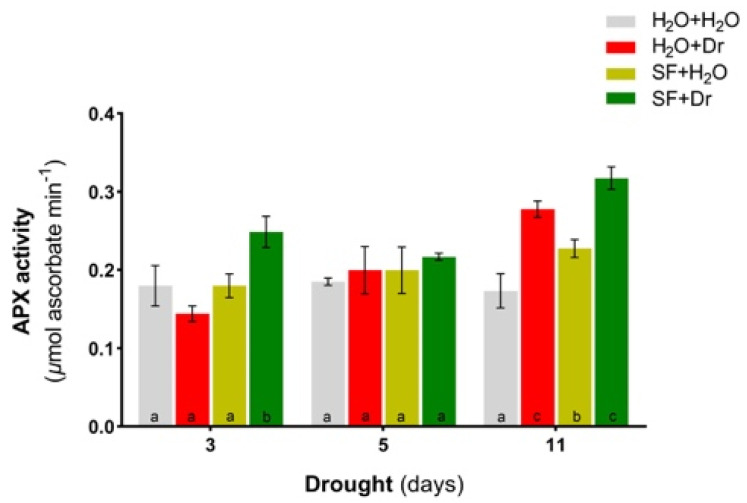
Ascorbate peroxidase activity in Arabidopsis leaves. Error bars denote the standard deviation (SD) of the mean. Different letters represent significant differences between the treatment means (*p* < 0.05, *n* = 3; one-way ANOVA, Tukey’s multiple comparison test). Dr, drought stress; SF, Super Fifty.

Next, APX activity was determined in plants at days 3, 5, and 11 of drought exposure. After 3 days, APX activity was significantly higher in SF-primed than nonprimed plants (Figure 4), followed by a trend of increased APX activity at day 11 of drought in SF-primed plants, indicating that APX contributes to ROS scavenging.

In addition, genes encoding proteins of the thioredoxin (TRX) superfamily were upregulated after SF treatment (SF+H_2_O and SF+Dr). TRXs are involved in the regulation of the cysteine/protein redox state in plants [66]. In particular, m-type TRXs are involved in photosynthetic activity, redox homeostasis and antioxidant mechanisms in plant plastids [67]. *TRXs* (m-types) are selectively upregulated after SF priming (Table 1).

Finally, several class III *PEROXIDASE* genes are downregulated by drought, a response largely mitigated by SF priming (Supplementary Figure S3a). In addition, the expression of other *PEROXIDASE* genes, in particular *PRX34*, was upregulated during drought, a response that was largely inhibited by SF priming (Supplementary Figure S3a). Cumulatively, our findings suggest an adjustment of the cellular redox status in SF-primed plants, enabling them to cope with otherwise adverse drought conditions.

### 2.7. SF Priming Alters Regulation of Drought Signaling at the Shoot Apical Meristem

Plants respond to drought stress by re-adjusting their physiology and stress-specific gene expression. *RD26* encodes a member of the plant-specific NAC transcription factor family [24] and is well-known for its responsiveness (enhanced expression) to plant dehydration, or treatment with ABA or H_2_O_2_ [24,25]. Throughout the life cycle of plants, the SAM ensures active growth and organ development and controls the transition from the vegetative to the reproductive stages [23]. Currently, it is not well-known how the SAM responds to drought stress and how it maintains its functionality to allow shoot growth during and after recovery from stress. The fact that SF-primed plants survived the drought stress prompted us to test whether the Arabidopsis SAM senses drought in SF-primed and unprimed plants. To this end, we performed a drought stress experiment (see Materials and Methods), harvested SAMs at 36 DAG (day 5 of drought), and 42 DAG (day 11 of drought), and tested *RD26* expression by RNA in situ hybridization.

**Figure 5:**
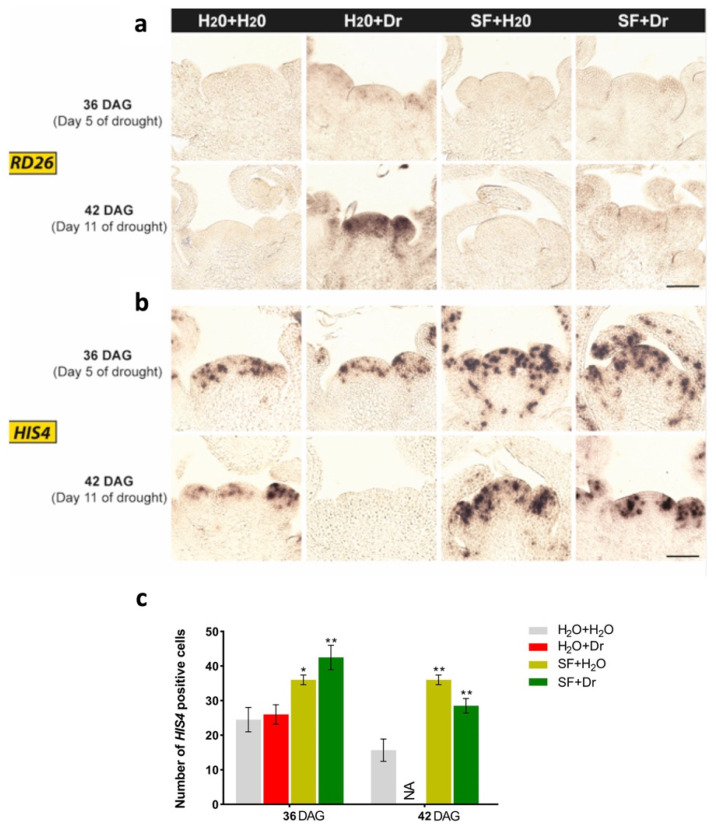
Expression of *RD26* and *HIS4* in the shoot apical meristems (SAMs) of SF-primed Arabidopsis plants. Expression of both genes was determined by RNA in situ hybridization. (**a**) Drought-induced expression of *RD26* in the SAMs of SF-primed and unprimed plants. Note the moderate expression of *RD26* in unprimed, drought-stressed plants at 36 DAG (days after germination; day 5 of drought) and the highly induced expression at 42 DAG (11 days of drought). (**b**) Expression of *HIS4* (marker of the S phase of the cell cycle) in the SAM. (**c**) Number of *HIS4*-positive cells in the SAMs of SF-primed and unprimed plants. In (**b**,**c**), note the absence (NA) of *HIS4*-positive cells in unprimed drought-stressed plants at 42 DAG (i.e., after 11 days of drought), but the high number of *HIS4*-positive cells in SAMs of SF-primed plants, even after extended drought. Error bars indicate standard deviation (*n* = 3). Statistical significance was calculated using Student’s *t*-test (* *p* < 0.05; ** *p* < 0.01). Dr, drought stress; SF, Super Fifty.

In the absence of SF priming, *RD26* expression was strongly induced by drought stress in the SAM (including vasculature and floral structures); *RD26* expression increased with time and after 11 days of drought it was considerably stronger than after 5 days (Figure 5a). This induction of *RD26* at the SAM was almost completely inhibited by SF priming (Figure 5a), consistent with the stress-mitigating effect of the biostimulant.

*RD26* expression is induced by H_2_O_2_ [24,68]. The enhanced expression of *RD26* during drought may, therefore, be associated with the elevated H_2_O_2_ level occurring in unprimed stressed plants. The protective role of SF priming by lowering H_2_O_2_ levels (see above) might explain the weak induction of *RD26* in the SAMs of primed plants.

### 2.8. SF Priming Keeps the Shoot Apical Meristem Functional During Drought

Since the growth parameters “final rosette diameter” and “leaf number” were significantly increased in SF-primed plants and virtually no induction of the stress marker gene *RD26* was observed at the SAM after SF priming, we monitored rosette growth in SF-primed and unprimed plants exposed to drought stress in more detail. As expected, drought significantly reduced rosette growth in unprimed plants (Supplementary Figure S4a), while SF-primed plants maintained their growth during drought and a recovery period after rewatering (Supplementary Figure S4a). In accordance with this, treatment with SF alone in the absence of drought stress did not affect leaf initiation rate (LIR) compared to control (H_2_O+H_2_O) plants indicating the absence of a toxic effect of SF at the concentrations applied (Supplementary Figure S4b). However, as expected, LIR was strongly reduced in drought-stressed plants (H_2_O+Dr). This strong inhibition of LIR was fully mitigated in plants primed with SF before the drought stress (SF+Dr; Supplementary Figure S4b).

As cell division at the SAM drives plant growth and development, we monitored expression of the core cell cycle marker gene *HIS4*, which labels the S-phase of the cell cycle [69], by RNA in situ hybridization. There was no detectable *HIS4* expression in the SAMs of drought-stressed plants at 42 DAG (11 days into drought; Figure 5b), suggesting that drought stress strongly inhibits cell division at the SAM, in accordance with the inhibition of shoot growth (Supplementary Figure S4a,b). However, *HIS4* was highly expressed in the SAMs of SF-primed plants, both under stressed and unstressed conditions, demonstrating that SF priming protects cell cycle progression from a permanent arrest (Figure 5b). The quantification of cells expressing *HIS4* at the SAM (Figure 5c) confirmed that SF priming protects the cell cycle from the lethal effects of drought stress.

In addition to *HIS4*, another cell cycle gene, *CYCLIN P2;1* (*CYCP2;1*), was strongly repressed by drought. This downregulation was completely reversed by SF priming (Table 1). Similarly, during drought stress, SF triggered an upregulation of *CYCLIN-DEPENDENT PROTEIN KINASE 3;2* (*CYCA3;2*) compared to drought-stressed plants not treated with SF. Collectively, our data thus demonstrate that SF priming positively affects plant growth and keeps the SAM functional.

### 2.9. SF Priming Induces Stomatal Closure

The observation of enhanced drought tolerance of SF-primed plants prompted us to analyze whether treatment with SF might affect stomatal behavior. To this end, we treated well-watered wild-type plants with SF and monitored stomatal aperture 2 and 4 h after the treatment. After 4 h, stomatal pores had lower apertures than pores from control plants not treated with SF (Supplementary Figure S5).

**Figure 6:**
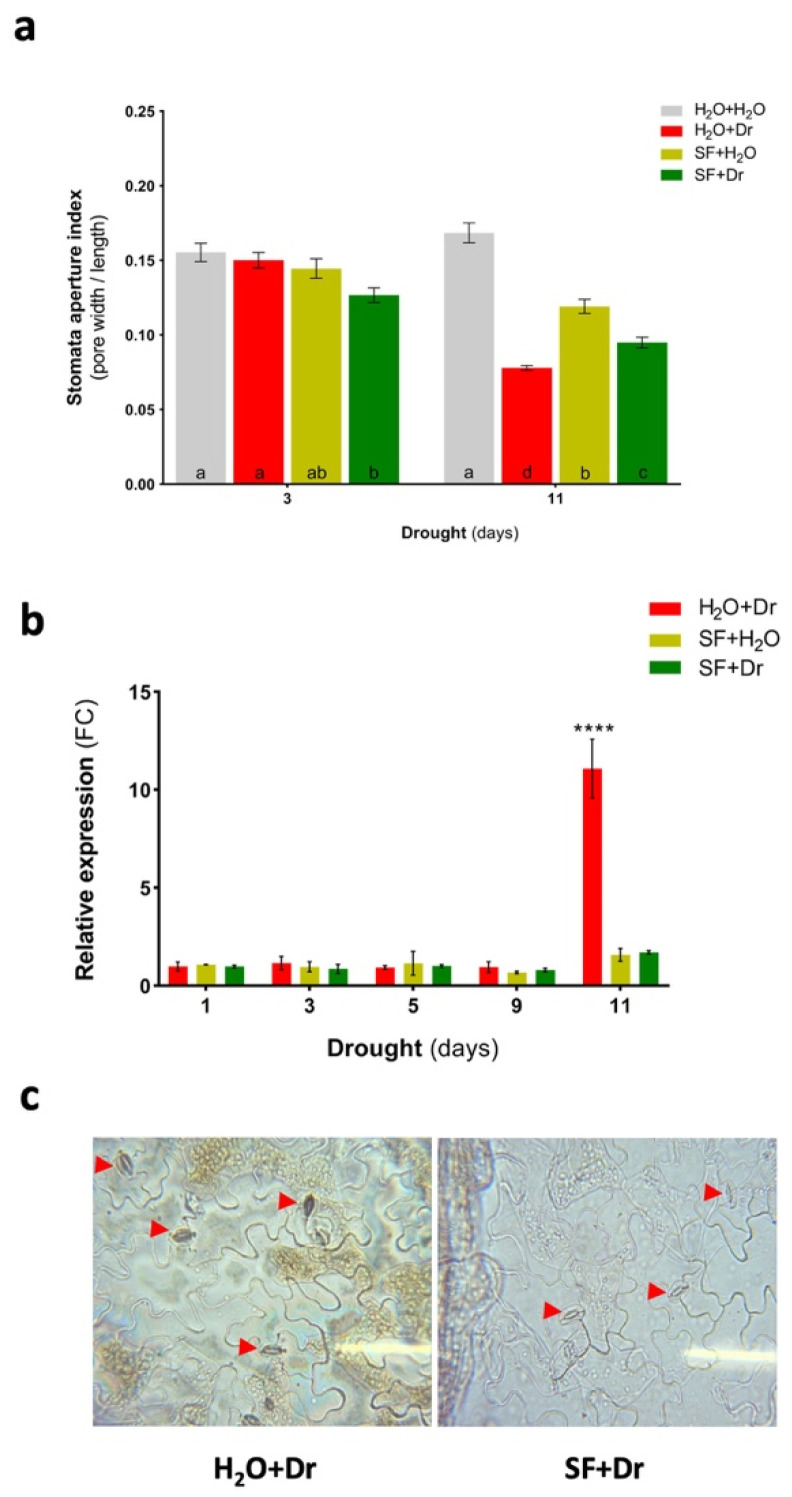
Stomatal closure response under drought. (**a**) Stomatal apertures in SF-primed and unprimed plants with the progression of drought stress. Note that, under well-watered conditions, SF priming (SF+H_2_O) leads to partial stomatal closure compared to nonprimed plants (H_2_O+H_2_O). Under conditions of drought stress, partial stomatal closure occurred earlier in SF-primed plants (SF+Dr) than in nonprimed plants (H_2_O+Dr). Error bars indicate standard error of the mean (SEM), *n* ≥ 250; treatments sharing different letters indicate significant differences from each other (*p* < 0.05; one-way ANOVA, Tukey’s multiple comparison test). (**b**) Relative expression of *PRX34* in SF-primed and unprimed plants. Values are expressed as fold change (FC) in expression normalized to those determined in unprimed, well-watered (H_2_O+H_2_O) controls. Asterisks represent significant differences in expression in unprimed drought-stressed (H_2_O+Dr) vs. primed (SF+Dr and SF+H_2_O) plants (*p* < 0.0001; one-way ANOVA, Tukey’s multiple comparison test). (**c**) Histochemical staining assay for ROS accumulation in leaf stomata. Leaf epidermal peels from plants (unprimed and primed) under drought stress were used for DAB staining following a reported protocol [74]. Red arrow heads indicate stomata. Dr, drought stress; SF, Super Fifty.

Next, the long-term effect of SF on stomatal aperture in the presence of drought stress was tested, at days 3 and 11 of drought exposure. After 11 days of drought, and in the absence of SF priming, stomatal pore size was strongly reduced compared to control, as expected (H_2_O+Dr vs. H_2_O+H_2_O). In well-watered conditions, SF priming already significantly reduced stomatal aperture compared to control (SF+H_2_O vs. H_2_O+H_2_O). This SF-induced stomatal closure was only slightly more enhanced during drought stress (Figure 6a).

We analyzed the expression of genes involved in ABA-/drought-mediated stomatal closure and whether this was modified by SF treatment. One of the genes affected by SF priming is *REGULATORY COMPONENTS OF ABA RECEPTOR 3* (*RCAR3*), which was downregulated in drought-stressed plants (H_2_O+Dr) compared to well-watered plants; this response was considerably decreased in drought-stressed plants previously subjected to SF priming (SF+Dr; Table 1; Supplementary Figure S3b). A supportive observation in this respect is that Arabidopsis plants overexpressing *RCAR3*, also known as *PYRABACTIN RESISTANCE-LIKE PROTEIN 8* (*PYL8*), are hypersensitive to ABA treatment, leading to reduced stomatal apertures, thereby contributing to reduced water loss [70].

OPEN STOMATA 1 (OST1) is a core signaling component involved in ABA-mediated stomatal closure; it acts upstream of ROS production in guard cells. The ABA-dependent accumulation of ROS by OST1 is achieved through the phosphorylation of two plasma membrane NADPH oxidases, also known as respiratory burst oxidase homologs (RbohF and RbohD in Arabidopsis) [41,71,72]. Our RNA-seq analysis revealed that *RBOHD* is downregulated by drought stress, a response not seen in SF-primed plants exposed to drought (Table 1). Thus, the SF priming-induced upregulation of *RCAR3* and *RBOHD* indicates an activation of a core ABA-dependent signaling pathway for stomatal closure in SF-primed plants subjected to drought stress.

Additionally, cytokinin-mediated regulation of guard cell ROS homeostasis affects stomatal closure [73]. In this mechanism, Arabidopsis Cytokinin Response Regulator 2 (ARR2) controls the expression of several apoplastic peroxidase genes (*PRX4*, *PRX33*, *PRX34*, and *PRX71*), thereby causing a cytokinin-mediated accumulation of H_2_O_2_ in guard cells and, therewith, stomatal closure during stress challenge. In the absence of SF priming, drought stress triggered a strong (118-fold) induction of *PRX34* expression, while in the presence of SF priming expression of *PRX34* increased by only 7.6-fold during the drought stress. Similarly, *ARR2* expression was induced 5.4-fold by drought stress in the absence of SF priming, which was completely mitigated by SF treatment prior to drought (Table 1). This pattern of *PRX34* and *ARR2* expression, as well as that of *PRX51* whose function has not been reported yet, was confirmed by quantitative real-time polymerase chain reaction (qRT-PCR; Supplementary Figure S3c–e). We also determined *PRX34* expression over the entire time frame of the drought stress experiment shown in Figure 6b, by qRT-PCR. While expression of *PRX34* remained largely unchanged until day 9 of the experiment, a strong increase in expression occurred at day 11 of the drought treatment in unprimed plants (H_2_O+Dr) compared to primed plants (SF+Dr; Figure 6b). Considering this, we determined the level of H_2_O_2_ in guard cells by DAB staining; after 11 days of drought, H_2_O_2_ level was high in guard cells of nonprimed plants, likely due to the high *PRX34* expression, but was much lower in guard cells of SF-primed plants (Figure 6c).

Thus, in addition to expressional changes of key ABA-dependent signaling components (*RCAR3* and *RBOHD*) we also observed discrete expression changes for *ARR2* and *PRX34* in SF-primed plants, which are important for cytokinin-mediated stomatal closure.

### 2.10. SF-Mediated Priming Likely Involves Multiple Signaling Pathways

**Figure 7:**
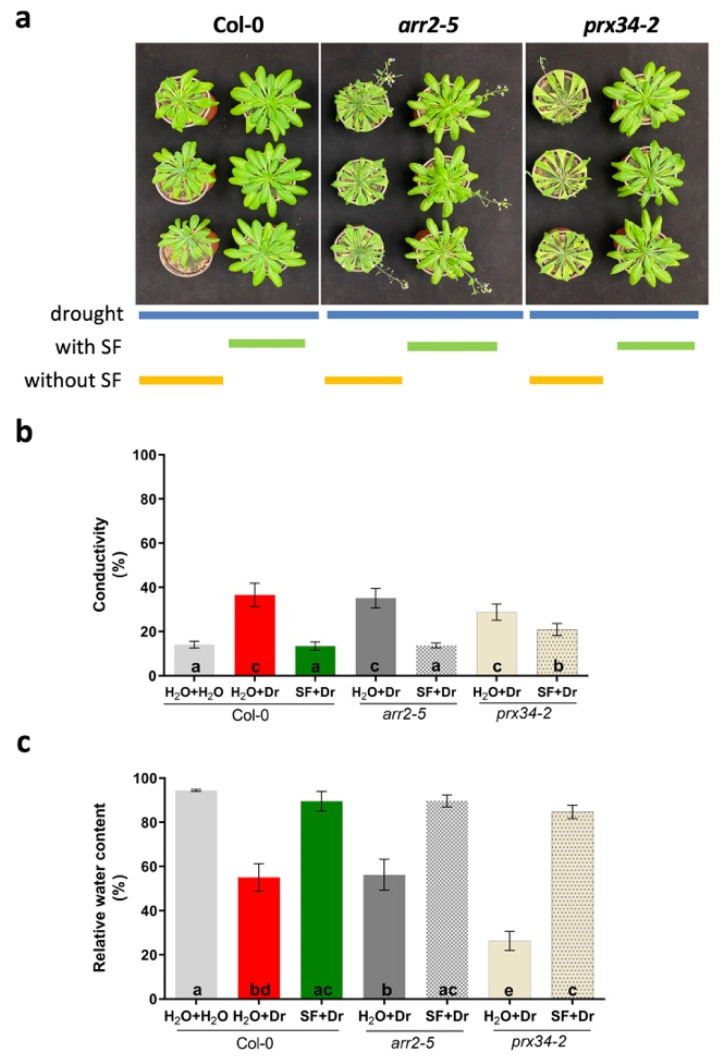
Response to drought in *arr2-5* and *prx34-2* mutants. (**a**) Response of plants exposed to 11 days of drought. (**b**) Ion conductivity as a measure of membrane damage. (**c**) Relative water content. Data are shown for wild type (Col-0), *arr2-5* and *prx34-2* plants (leaves 10 and 11). Treatments sharing different letters represent statistically significant differences from each other (*p* < 0.05; one-way ANOVA, Tukey’s multiple comparisons test). Dr, drought stress; SF, Super Fifty.

The function of *ARR2* and *PRX34* in stomatal closure as a response to drought in SF-primed and -unprimed plants was assessed using Arabidopsis mutants (T-DNA insertion lines) deficient in *ARR2* (*arr2-5*) or *PRX34* (*prx34-2*; Figure 7). qRT-PCR results revealed strongly reduced expression of the respective genes as expected for loss-of-function mutations (Supplementary Figure S6) [73]. The *arr2-5* and *prx34-2* knockout lines were exposed to drought for 11 days. Compared to wild type (WT, Col-0), *arr2-5* phenotypically showed increased sensitivity towards drought stress (enhanced leaf wilting; Figure 7a) although ion conductivity and RWC were not detectably different between the lines (Figure 7b,c). Likewise, *prx34-2* showed increased sensitivity towards drought compared to WT (increased wilting; Figure 7a), which was accompanied by a significantly lower RWC, but no detectable change in ion leakage at day 11 (Figure 7b,c). Considering the results obtained, we conclude that both *ARR2* and *PRX34* function in drought-mediated stomatal closure; their loss of function reduces the capacity of plants to withstand drought stress, likely due to increased water loss. In accordance with this, previous studies have shown that *arr2-5* and *prx34-2* mutants exhibit a reduced stomatal response to cytokinin treatment [73].

Next, it was assessed whether SF priming depends on *ARR2* and *PRX34* during drought. To this end, *arr2-5* and *prx34-2* mutants were primed with SF and then exposed to drought stress. We found that both mutants, when primed with SF, were able to withstand the drought. While ion conductivity (hence, ion leakage due to membrane damage) under drought was similar in the *arr2-*5 mutant and WT, it was more prominent in *prx34-2* compared to WT (Figure 7b). With respect to RWC, no significant difference was observed between the two mutants and the WT under priming conditions (Figure 7c), demonstrating that SF priming is not solely dependent on ARR2- and PRX34-mediated stomatal closure but involves multiple mechanisms to enhance drought tolerance.

## 3. Discussion

Biostimulants can enhance the tolerance of plants to abiotic stresses and improve productivity and crop quality [22]. We recently demonstrated that a specific extract of *A. nodosum*, Super Fifty (SF), reduces paraquat-induced oxidative stress in *Arabidopsis thaliana* and vegetable crops [12,75]. SF is a concentrated alkaline extract of the seaweed *A. nodosum,* which contains a range of unique carbohydrates such as fucoidan, alginate, and laminarin, as well as proteins and minerals [14]. Studies in plants have shown that certain chemicals and the exposure to mild abiotic cues can trigger a process known as “priming” to induce robust and efficient defense mechanisms upon a subsequent stress challenge [76,77]. This study was based on the hypothesis that pretreating plants with the *A. nodosum* extract SF can prime and induce tolerance to drought stress.

Drought stress typically inhibits plant growth [78,79,80,81]. The results presented here demonstrate that SF protects Arabidopsis plants from the otherwise deleterious effects of drought and enables them to maintain growth during stress (Supplementary Figure S4). SF also reduces the accumulation of toxic ROS levels in leaves. Furthermore, SF priming helps plants to retain their leaf initiation speed, and primed plants are able to maintain leaf growth rate and increase in rosette size during drought stress, which otherwise is affected in unprimed control plants (H_2_O+Dr) (Supplementary Figure S4a,b). Whole transcriptome analysis identified the activation of distinct signal transduction networks and a core ABA-dependent signaling pathway concomitant with an elevated drought stress response in SF-primed plants. This analysis, combined with RNA in situ hybridization, revealed that the dehydration- and H_2_O_2_-induced NAC transcription factor *RD26* [24,68], which is involved in abiotic stress signaling [25], is strongly upregulated in the SAMs of drought-stressed plants (H_2_O+Dr). Of importance in this respect is that overexpression of *RD26* in transgenic plants leads to reduced plant growth and a modified response to brassinosteroids (BR) in Arabidopsis [82]. Moreover, during unfavorable conditions such as drought, a growth-promoting and BR-regulated transcription factor, BRI1-EMS SUPPRESSOR 1 (BES1), is degraded, causing the plants to shut down (i.e., limit) their growth [83]. Interestingly, SF priming strongly suppressed *RD26* expression in the SAM (Figure 5a) and maintained *BES1* expression levels under drought (Table 1). This suggests that SF priming targets and modulates transcription regulators that are key for coordinating growth with stress responses in Arabidopsis.

In addition, cell cycle genes such as *CYCP2;1*, *CYCA3-2*, and *HISTONE H4* are upregulated in primed plants (SF+Dr) during drought. Cyclin-dependent kinase (CDK)–cyclin (CYC) complexes are the core cell cycle regulators and phosphorylate a variety of substrates to permit the orderly progression through the cycle phases [84]. *CYCP2;1* is a cyclin gene involved in the integration of genetic and nutritional information to promote meristem cell division in Arabidopsis [85]. The *CYCA3;2* gene encodes a cyclin-dependent protein kinase that controls cell proliferation in meristems [86]. The induction of these cell cycling genes in SF-primed plants may play a role in priming-induced protection and maintenance of cell growth under stress.

This conclusion is supported by RNA in situ hybridization which demonstrated expression of *HISTONE H4* (*HIS4*) at the SAMs of Arabidopsis plants. *HIS4* is a G1-S phase cell cycle marker gene and functions in cell division. *HIS4* expression was strongly active at the SAMs of SF-primed plants (both stressed and unstressed) (Figure 5b,c), while in the absence of SF priming, no *HIS4* expression was detectable in drought-stressed plants (H_2_O+Dr) after 11 days of withholding irrigation. Maintenance of the cell cycle by sustained meristematic activity is crucial for plant survival under stress [87]. Our results clearly demonstrate that priming with SF maintains cell cycle progression at the SAM during drought stress, thereby keeping the SAM in a functional state.

The transcriptome analysis also revealed that *PEROXIDASE34* (*PRX34*), a ROS (H_2_O_2_) generator, and other genes of the same family are strongly induced by drought in unprimed plants (H_2_O+Dr; Supplementary Figure S3a). This induction was considerably suppressed by SF priming. Moreover, other genes known for their role in cellular oxidative burst, such as *OXIDATIVE SIGNAL-INDUCIBLE1* (*OXI1*) and *RESPIRATORY BURST OXIDASE HOMOLOG C* (*RBOHC*, *RHD2*), were suppressed in SF-primed plants under drought (SF+Dr). *OXI1* encodes a serine/threonine protein kinase of the AGC kinase family and is induced by H_2_O_2_ and various elicitor treatments [88]. The kinase mediates oxidative burst and ROS-dependent cellular responses [56]. Likewise, RbohC is an NADPH oxidase involved in ROS production [89,90]. *RBOHC*, along with other *RBOH* genes, shows increased expression during drought and salt stress [91]. Similarly, induction of these oxidative burst-related genes in drought-stressed plants occurred in the absence of SF priming (H_2_O+Dr), while SF priming strongly suppressed their expression during drought. The physiological roles of *OXI1* and *RBOHC*, as well as the associated ROS burst during drought, may explain the higher oxidative damage in unprimed plants which is rescued by SF priming. These results clearly show that priming with SF reduces drought stress-induced oxidative damage by minimizing cellular oxidative burst in primed plants.

SF induces genes coding for enzymatic antioxidants linked to the ascorbate-glutathione cycle. This is in line with our previous findings that strongly suggested an involvement of the ascorbic acid-associated ROS detoxification pathway for reducing oxidative stress in Arabidopsis [12]. The ascorbic acid (AsA)–glutathione (GSH) pathway, also known as the Asada–Halliwell pathway, plays a vital role in detoxifying abiotic stress-induced ROS [59]. Furthermore, we observed that drought stress-induced repression of the antioxidant gene *APX4* is mitigated by SF priming (Table 1); similarly, SF eliminates the effect of oxidative stress induced by paraquat treatment [12]. Our study also indicated increased ascorbate peroxidase activity and reduced H_2_O_2_ accumulation in SF-primed drought-stress plants. We conclude that APX4, a known H_2_O_2_ scavenger [61], may have an important role in the SF priming-induced ROS detoxification process.

Intercellular regulation in response to environmental stress is achieved via a complex chain of signals that eventually affect all plant organs. For example, transpiration through stomata is remotely controlled by drought-triggered root-to-shoot signaling [92]. Their role is to control the flow of gases between plants and the surrounding environment [93]. Under drought, the phytohormone ABA induces stomatal closure and the expression of stress-responsive genes [94,95]. However, guard cell opening or closure is far more complex than originally thought and can be affected by several additional regulatory components involving a complex network of genes [92,96].

SF leads to a prompt reduction in stomatal aperture after 4 h (Supplementary Figure S5), and after 3 days of drought stress, SF-primed plants are able to reduce their stomatal aperture more than unprimed plants (Figure 6a). Thus, the faster response of the SF-primed plants to the initial stages of drought stress may contribute to the outstanding preservation of their water contents during the longer drought exposure (11 days), in contrast with the severe wilting of drought-stressed unprimed plants after prolonged drought stress. The prompt stomatal closure induced by SF priming not only preserved water content in leaves under drought stress, but also reduced membrane damage, as reflected by reduced ion leakage. Whole transcriptome level analysis revealed that a core ABA-dependent signaling pathway is activated to induce drought stress responses in SF-primed plants. Specific transcript level modulation of genes coding for RCAR3 and RBOHD, related to ABA-dependent signaling pathways for stomatal closure, was activated in SF-primed plants exposed to drought (Table 1). Thus, it can be deduced that SF priming represents a promising strategy to maintain high leaf water content and will serve as a practical management tool to mitigate the adverse impact of drought. 

In addition, while drought stress led to H_2_O_2_ accumulation in guard cells of nonprimed plants, this increase in H_2_O_2_ was not observed in SF-primed plants, likely due to the repressed induction of *PRX34* expression after SF treatment. We found that *arr2-5* and *prx34-2* knockout plants are sensitive to water-deficit conditions; mutants primed with SF restored their ability to withstand the drought (Figure 7a–c), suggesting that SF priming-induced tolerance involves additional genes and regulatory elements.

In summary, this study demonstrates that prior application of SF as a priming agent can confer drought tolerance to plants. SF-primed plants showed a transcriptional reconfiguration and physiological adjustments which enhance drought tolerance.

## 4. Materials and Methods

### 4.1. General

*Arabidopsis thaliana* (L.) Heynh., accession Columbia-0 (Col-0), was used as wild type (WT). The T-DNA insertion mutants *arr2-5* (GK-269G01) and *prx34-2* (GK-728F08) [73] were obtained from the Nottingham Arabidopsis Stock Centre (http://arabidopsis.info; Loughborough, UK). Oligonucleotides used for genotyping and qRT-PCR (Supplementary Table S1) were obtained from Eurofins MWG Operon (Ebersberg, Germany). Biostimulant SF was obtained from BioAtlantis Ltd. (Tralee, Ireland). DNA sequencing was performed by LGC Genomics (Berlin, Germany). The Arabidopsis Information Resource (TAIR; https://www.arabidopsis.org; Newark, CA, USA) was used to obtain gene and full-length coding sequences (CDS). Sequence analyses were performed using tools available at the National Center for Biotechnology Information (https://www.ncbi.nlm.nih.gov) and JustBio (http://www.justbio.com).

### 4.2. Growth Conditions and Physiological Experiments

Plants were grown in soil in pots (13 cm diameters) in day-neutral photoperiod (12 h light/12 h dark) with 22 °C/18 °C, and 65–70% relative humidity. Plants were grown in a mixture of potting soil (Einheitserde GS90; Gebrüder Patzer, Sinntal, Germany), and vermiculite mixed in a 2:1 (*v*/*v*) ratio. Plants were sprayed with SF at a concentration of 0.2% (*v*/*v*), starting 4 h after day light and the foliar application was repeated 2 days after the first spray (i.e., 1st and 2nd applications on 25 and 27 DAG, respectively; Supplementary Figure S1). Spraying was applied from the top, from a 15 cm distance, and special care was taken to ensure that fine mist of spray covered whole leaves to avoid foliage overdose. A constant supply of irrigation water was maintained and 3 days after the second SF application, plants were exposed to drought stress. Before starting the drought stress, pots were fully saturated with water and then were exposed to stress by stopping the irrigation. Stress-related parameters such as leaf wilting symptoms were visually observed and the number of rosette leaves and the rosette diameters were recorded. For measuring rosette diameter, data were recorded from six plants per treatment (*n* = 6), and images were analyzed using ImageJ (https://imagej.nih.gov/ij; Bethesda, MD, USA). For the determination of rosette leaf number, data were recorded from eight plants per treatment (*n* = 8). Both above experiments were performed at 40 DAG. Ion leakage and relative water content (RWC) of leaves were measured to set-up critical time points where primed plants significantly tolerated the stress period compare to control untreated (i.e., unprimed) plants. For measuring ion leakage, leaf samples were collected from six plants per treatment (*n = 6*). For RWC, leaf fresh weight, saturated/turgid leaf weight, and dry weight were calculated according to the method described [97], and finally RWC was calculated using the formula “RWC (%) = [(W − DW)/(TW − DW)]*100”, with the following parameters: W, sample fresh weight; DW, sample dry weight; TW, sample turgid weight. Leaf samples were collected from four plants per treatment (*n* = 4) for the RWC data. Plants were randomized each week to reduce variability. The experiments above were repeated with three biological replicates.

### 4.3. Drought Treatments and Sampling for Whole-Genome Transcriptome

A schematic presentation of SF priming and drought treatments is given in Supplementary Figure S1. Soil pots were weighed to ensure equal amounts of soil in each pot (90 g). Three-week-old Col-0 plants were treated twice with SF; the first treatment was carried out at 25 DAG, and the second at 27 DAG. The SF treatment was omitted in unprimed plants (H_2_O+H_2_O, H_2_O+Dr). Plants were then subjected to drought stress from 31 DAG until 42 DAG (H_2_O+Dr, SF+Dr). For performing drought treatment, pots were fully watered on 30 DAG, but no more at 31 DAG until 42 DAG (i.e., 11 days of drought). A constant supply of irrigation water was maintained in unstressed control treatments (H_2_O+H_2_O, SF+H_2_O) to ensure 100% water holding capacity. Soil moisture was monitored throughout the experiment and drought treatments were stopped at 42 DAG (day 11 of drought) when 10% water holding capacity was confirmed (Supplementary Figure S7). Leaf samples for RNA extraction were collected at 42 DAG from six plants per treatment (*n = 6*), pooled, and immediately frozen in liquid nitrogen. The experiment was performed with three biological replicates.

### 4.4. Analysis of Growth Parameters

SF priming and drought experiments were performed as described above. For measuring rosette area, images of full rosettes were taken at 10 DAG until the flowering time point (48–50 DAG, depending on the bolting time) at 5-day intervals. Images from at least 18 plants per replicate (three replicates in total) were analyzed using ImageJ software (Bethesda, MD, USA). The leaf initiation rate was determined as reported [98].

### 4.5. Determination of ROS Levels

Rosette leaves were harvested at 42 DAG, immediately frozen in liquid nitrogen, and H_2_O_2_ level was determined using an Amplex Red hydrogen peroxide/peroxidase assay kit (Invitrogen; Paisley, UK) as reported [30]. Briefly, samples were ground in liquid nitrogen, and 30 mg of ground frozen tissue was placed in an Eppendorf tube and kept frozen. Then, 400 mL 20 mM sodium phosphate buffer, pH 7.4, was immediately added to the tube and mixed. The extraction was centrifuged at 10,000× *g* for 15 min at 4 °C, and the supernatant was used for the assay. Measurements were performed at excitation and emission wavelengths of 560 and 590 nm, respectively, using a 96-well Tecan F200 Infiniti Multi-mode plate reader (TECAN, Männedorf, Switzerland). H_2_O_2_ levels are given in pmol of H_2_O_2_/mg frozen tissue. For in planta H_2_O_2_ detection, 3,3-diaminobenzidine (DAB) staining was performed as described [99]. Leaves were detached and immediately vacuum-infiltrated with 1 mg/mL DAB dissolved in 50 mM Tris-acetate (pH 5.0) for 6 h. For visualization of H_2_O_2_, chlorophyll was removed by heating to 90 °C in bleaching solution (lactic acid:glycerol:ethanol, 1:1:4) for 15 min. Arabidopsis leaf epidermal peels were prepared as described [100]. Histochemical staining of leaf epidermal peels of the detection of ROS was performed as previously described [74].

### 4.6. Transcriptome Sequencing and Data Analysis

For transcriptome sequencing, total RNA was extracted from three replicates (six plants each) for each treatment using the Trizol method, and purification was carried out using the Ambion PureLink RNA Mini Kit (Thermo Fisher Scientific, Waltham, MA, USA) according to the manufacturer’s instructions. mRNA was prepared by LGC Genomics using oligo (dT) magnetic beads, followed by cDNA synthesis. Sequencing libraries were prepared using the Ovation Rapid Library Preparation kit (NuGEN, San Carlos, CA, USA). The libraries were sequenced using the Illumina HiSeq 4000 platform (Illumina Inc., San Diego, CA, USA) to obtain 75 bp-long single-end reads. For data analysis, sequencing adaptors were trimmed from raw reads using cutadapt and reads with final lengths of <20 were discarded. Ribosomal RNA contamination was removed using SortMeRNA (v2.1) [101], and reads aligning to rRNA were filtered out. Filtered reads for all samples were quantified using kallisto (v0.43.0; bootstraps: 100) [102] against Arabidopsis cDNA sequences (Araport11) [103]. Differential expression analysis was carried out using the EdgeR package in R/Bioconductor [104]. Significantly differentially expressed genes were identified using log2 fold change ≥1 and FDR cut-off of <0.001. GO enrichment was carried out using the GOSeq R-package [105], with FDR cut-off of ≤0.1.

### 4.7. Quantitative Real-Time Polymerase Chain Reaction

Total RNA was extracted using the Trizol method and purification was carried out using the Ambion PureLink RNA Mini Kit (Thermo Fisher Scientific; Waltham, MA, USA) according to the manufacturer’s instructions. Quantitative real-time PCR was performed in 96-well plates using an ABI PRISM 7900HT sequence detection system (Applied Biosystems, Dreieich, Germany). All reactions were performed in triplicates using SYBR Green-PCR Master Mix (Applied Biosystems, Darmstadt, Germany) and gene-specific primers listed in Supplementary Table S1. Expression data were normalized against *ACTIN2* as a reference gene using the ∆∆*C*_t_ method [106].

### 4.8. Enzyme Assay

Ascorbate peroxidase (APX) activity was assayed following a reported protocol [107] with slight modifications, as described [108]. Briefly, approximately 80 mg of ground leaf sample was homogenized in 1 mL ice-cold 50 mM potassium phosphate buffer (pH 7.0) containing 1% (*w*/*v*) polyvinylpyrrolidone (PVP), 0.2 mM EDTA, and 0.1% (*v*/*v*) Triton X-100. The reaction mixture for determining APX activity contained 50 mM potassium phosphate buffer (pH 7.0), 0.1 M ascorbate, and 10 mM H_2_O_2_. Enzyme activity was determined by measuring the oxidation rate of ascorbate at 290 nm (absorbance coefficient of 2.8 mM^−1^ cm^−1^).

### 4.9. Determination of Stomatal Aperture

Stomata imprints from young leaf epidermal peels were collected using the dental resin impression method [109]. Briefly, President Light Body polyvinylsiloxane dental resin (REF 60019938, Type 3, low consistency; Coltène/Whaledent AG, Altstätten, Switzerland) was placed on a microscope slide and newly detached leaves were placed on the fresh resin on their abaxial sides. Leaves were then removed from the compact resin and the dental resin mold was filled with nail polish to create a cast that was examined by microscopy. Imprint samples were collected from leaves of 40 plants per treatment and time point (i.e., 2 and 4 h), and randomly selected leaf areas were analyzed in each case. Stomatal aperture sizes of at least 250 stomata were determined using ImageJ software (https://imagej.nih.gov/ij; Bethesda, MD, USA). The widths and lengths of stomata pore apertures were recorded and the stomata aperture index (SAI) was calculated by dividing aperture width by aperture length [110].

### 4.10. RNA In Situ Hybridization

Sample preparations including harvesting, fixing and embedding were performed as described [98]. Briefly, meristems of Arabidopsis (Col-0) plants were harvested, fixed and embedded in wax using an automated tissue processor (ASP200S; Leica, Wetzlar, Germany) and embedding system (HistoCore Arcadia; Leica, Wetzlar, Germany). Tissue sections of 8 μm thickness were prepared using a Leica RM2265 rotary microtome. Hybridization probes were synthesized using a Digoxigenin RNA Labelling kit (Roche, Mannheim, Germany) employing PCR products of whole open reading frames of the target genes. RNA in situ hybridizations were performed as described [111]. Primer sequences used in this analysis are given in Supplementary Table S1.

### 4.11. Statistics

The program GraphPad Prism 8 (www.graphpad.com; GraphPad Software, San Diego, CA, USA) was used for statistical analysis and data presentation using Student’s *t*-test and one-way ANOVA followed by Tukey’s and Dunnett’s multiple comparisons test to adjust the *p*-values.

### 4.12. Gene Codes

Arabidopsis gene codes are: ACTIN2, AT3G18780; HISTONE H4, AT2G28740; PEROXIDASE 34, AT3G49120; ARABIDOPSIS RESPONSE REGULATOR 2, AT4G16110; RESPONSIVE TO DESICCATION 26, AT4G27410. Additional gene codes are given in Table 1 and Supplementary Files S1–S3.

### 4.13. Data Availability

The RNA-sequencing data can be retrieved from NCBI under the BioProject ID PRJNA592062.

## 5. Conclusions

We demonstrated that a specific biostimulant produced from the seaweed *A. nodosum*, Super Fifty (SF), acts as a molecular priming tool to fine-tune growth and drought stress tolerance in plants. SF priming significantly improves the functionality of the shoot apical meristem under drought stress which is vital for plant growth. It also enhances relative water content in leaves during drought, concomitant with a reduction in cell membrane damage. A major mechanism by which SF achieves these effects may be by lowering the cellular level of toxic ROS—in particular H_2_O_2_. SF-based molecular priming has the potential to improve resistance of crops to challenges arising from climate change.

## Figures and Tables

**Table 1:** Super Fifty and drought alter the expression of genes representing the activation of drought signaling pathways for stress tolerance. Values are trimmed means of M-values (TMMs) averaged across three biological replicates. Dr, drought stress; SF, Super Fifty.

AGI Code	Gene Name	Description/Function	Expression Values (TMM)			
			Untreated (H_2_O+H_2_O)	Drought-Stressed (H_2_O+Dr)	SF-Primed (SF+H_2_O)	SF-Primed+Drought-Stressed (SF+Dr)
**Genes induced by SF priming in the absence of drought stress**						
AT2G29460	*GSTU4*	Glutathione S-transferase tau 4	1.49	605.19	20.11	23.76
AT2G29350	*SAG13*	Senescence-associated gene 13	3.69	664.02	85.49	35.16
AT5G40390	*RFS5, SIP1*	Raffinose synthase family protein	1.08	92.61	8.01	25.74
AT1G52690	*LEA7*	Late embryogenesis abundant protein	2.42	6582.52	54.96	123.57
AT5G06760	*LEA4-5, LEA46*	Late embryogenesis abundant 4–5 protein	0.31	2817.88	35.40	300.14
AT2G35980	*YLS9*	Yellow-leaf-specific gene 9	0.05	6.90	1.06	0.70
AT2G42560	*LEA25*	Late embryogenesis abundant protein	0.04	418.16	6.80	1.38
AT3G02480	*ABR*	Late embryogenesis abundant protein	15.41	4582.45	105.24	138.61
AT3G44300	*NIT2*	Nitrilase 2	0.84	416.61	101.37	55.04
AT2G37180	*PIP2-3*, *RD28*	Aquaporin-like superfamily protein	17.92	6.69	58.51	27.92
AT2G37170	*PIP2B*	Plasma membrane intrinsic protein 2	66.91	11.31	171.42	143.84
AT4G12490	*AZI3*	Lipid-transfer protein	0.04	0.64	6.88	2.73
AT4G12480	*EARLI1*	Lipid-transfer protein (putative)	0.70	0.45	8.00	1.93
**Genes involved in ABA signaling**						
AT4G03415	*PP2C52*	Protein phosphatase 2C family protein	27.41	5.66	24.75	13.91
AT4G26080	*ABI1*	Protein phosphatase 2C family protein	21.23	557.80	57.47	311.62
AT5G57050	*ABI2*	Protein phosphatase 2C family protein	2.94	308.02	11.87	90.17
AT2G29380	*HAI3*	Highly ABA-induced PP2C protein 3	0.07	73.94	0.53	0.23
AT1G78290	*SNRK2.8*, *SRK2C*	Protein kinase superfamily protein	3.36	1.25	6.24	10.67
AT4G34000	*ABF3*	Abscisic acid-responsive elements-binding factor 3	2.24	32.19	5.66	66.94
AT5G47910	RBOHD	Respiratory burst oxidase homologue D	19.56	11.63	24.85	31.15
AT5G53160	*PYL8, RCAR3*	Regulatory components of ABA receptor 3	194.42	21.54	228.40	87.45
AT3G11410	*PPT2CA*	Protein phosphatase 2CA	70.86	1061.27	131.39	848.51
AT4G25480	*DREB1A*	Dehydration response element binding B1A	0.04	1.99	0.20	6.42
AT5G11590	*DREB3*, *TINY2*	Dehydration response element binding B3	1.70	0.63	1.50	14.27
AT5G47640	*NF-YB2*	Nuclear factor Y, subunit B2	52.72	350.49	81.55	1474.77
AT4G14540	*NF-YB3*	Nuclear factor Y, subunit B3	86.44	20.03	104.90	108.38
AT5G06510	*NF-YA10*	Nuclear factor Y, subunit A10	2.36	6.64	2.64	11.46
**Genes linked to ERF53-dependent transcription**						
AT2G20880	*ERF53*	AP2/ERF transcription factor 53	0.15	1.23	4.82	6.85
AT3G01650	*RGLG1*	RING domain ligase 1	4.16	60.77	4.46	4.13
AT5G14420	*RGLG2*	RING domain ligase 2	11.96	36.40	12.06	10.88
AT5G47220	*ERF2*	AP2/ERF transcription factor 2	11.75	6.85	16.90	29.06
AT4G28140	*ERF54*	AP2/ERF transcription factor 54	0.83	6.98	13.39	23.40
AT1G06160	*ERF94*, *ORA59*	Octadecanoid-responsive AP2/ERF transcription factor 59	2.58	0.10	4.82	4.95
**Genes involved in antioxidant ascorbate glutathione cycle and ROS homeostasis**						
AT4G09010	*APX4*	Ascorbate peroxidase 4	117.31	37.68	96.79	137.32
AT1G77490	*APXT*, *TAPX*	Thylakoidal ascorbate peroxidase	43.36	9.34	48.15	71.70
AT5G16710	*DHAR3*	Dehydroascorbate reductase 1	216.89	94.41	215.00	339.78
AT2G25080	*GPX1*	Glutathione peroxidase 1	161.75	78.19	168.16	171.16
AT3G55040	*GSTL2*	Glutathione transferase lambda 2	130.57	68.16	151.44	192.82
AT1G03680	*ATHM1*, *THM1*	Thioredoxin M-type 1	517.98	248.21	601.57	842.87
AT3G15360	*ATHM4*, *TRX-M4*	Thioredoxin M-type 4	354.88	254.22	394.58	596.43
AT1G28480	*GRX480*, *GRXC9*	Thioredoxin superfamily protein	0.61	2.20	1.26	7.46
AT1G50320	*ATHX*, *THX*	Thioredoxin X	358.33	230.93	425.76	532.61
AT3G11630	*BAS1*	Thioredoxin superfamily protein	527.03	153.38	541.26	712.82
AT3G26060	*ATPRX Q*, *PRXQ*	Thioredoxin superfamily protein	239.96	62.0	251.24	333.39
AT4G03520	*ATHM2*	Thioredoxin superfamily protein	513.22	324.57	578.12	760.03
AT4G15660	*GRXS8*	Thioredoxin superfamily protein	32.06	4.34	26.35	21.11
**Genes involved in cytokinin-mediated stomatal closure**						
AT3G49120	*PRX34*	Peroxidase 34	50.30	5938.87	258.46	381.85
AT4G16110	*ARR2*	Response regulator 2	3.39	18.33	3.67	3.29
AT3G25250	*OXI1*	Oxidative signal-Inducible 1	0.06	1.97	0.25	0.29
AT5G51060	*RBOHC*, *RHD2*	Respiratory Burst Oxidase Homolog C	0.06	2.51	0.16	0.04
**Genes involved in growth regulation and cell cycling**						
AT3G21870	*CYCP2;1*	Cyclin P2;1	3.57	0.95	8.87	16.75
AT1G47210	*CYCA3;2*	Cyclin-dependent protein kinase 3;2	4.35	4.54	4.22	14.86
AT2G28740	*HISTONE H4*	Histone H4 (HIS4)	38.66	29.96	29.51	72.47
AT4G27410	*RD26*	Responsive to desiccation 26	6.33	1086.04	37.74	313.78
AT1G19350	*BES1*	BRI-EMS suppressor 1	131.59	27.67	148.71	135.43

## Supplementary Materials

The following are available online at https://www.mdpi.com/1422-0067/22/3/1469/s1.
