# Supplementary material for: Priming with a Seaweed Extract Strongly Improves Drought Tolerance in Arabidopsis
Source: Int J Mol Sci. 2021 Feb 2;22(3):1469. doi: 10.3390/ijms22031469 (PMC7867171; doi:10.3390/ijms22031469)
Supplement: Supplementary file 1 [file ijms-22-01469-s001.zip › Suppl files/Suppl. Figures_31.01.2021.pdf]

Supplementary Figure S1

SF-primed and drought-stressed (SF+Dr)

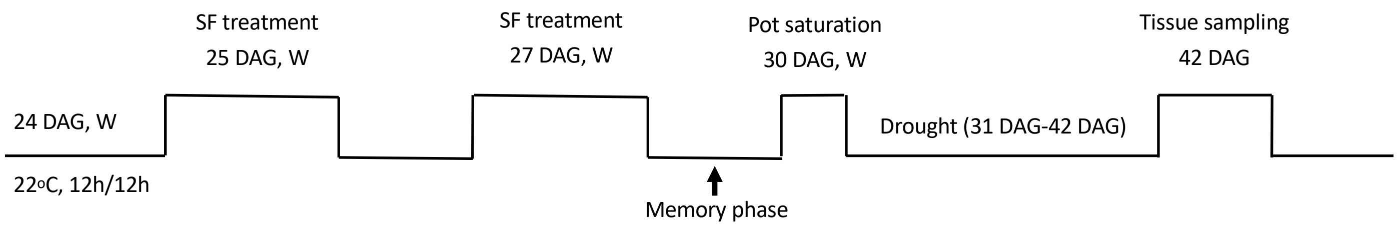

Unprimed and drought-stressed (H_2O+Dr)

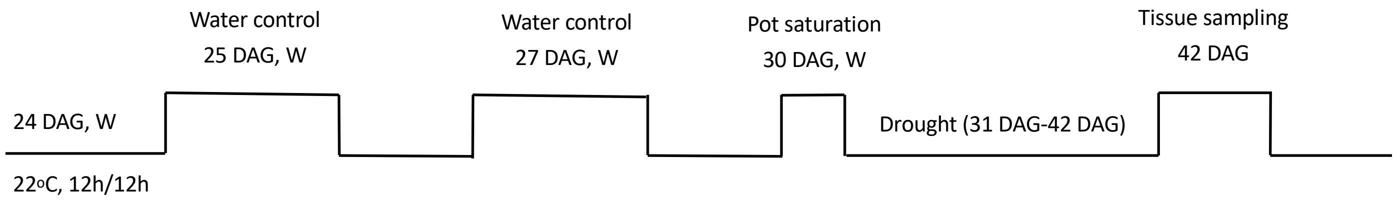

Supplementary Figure S1. Schematic representation of the SF priming and drought experimental set-up. Unless otherwise indicated, 25-day-old Arabidopsis Col-0 plants were subjected to Super Fifty (SF) treatment by foliar application on leaves. SF treatment was repeated at 27 days after germination (DAG). SF treatment was omitted in control (unprimed) plants. Once primed (three days after the SF treatment), plants were exposed to drought stress by stopping irrigation for few consecutive days, and plant phenotypes were observed.

Supplementary Figure S2

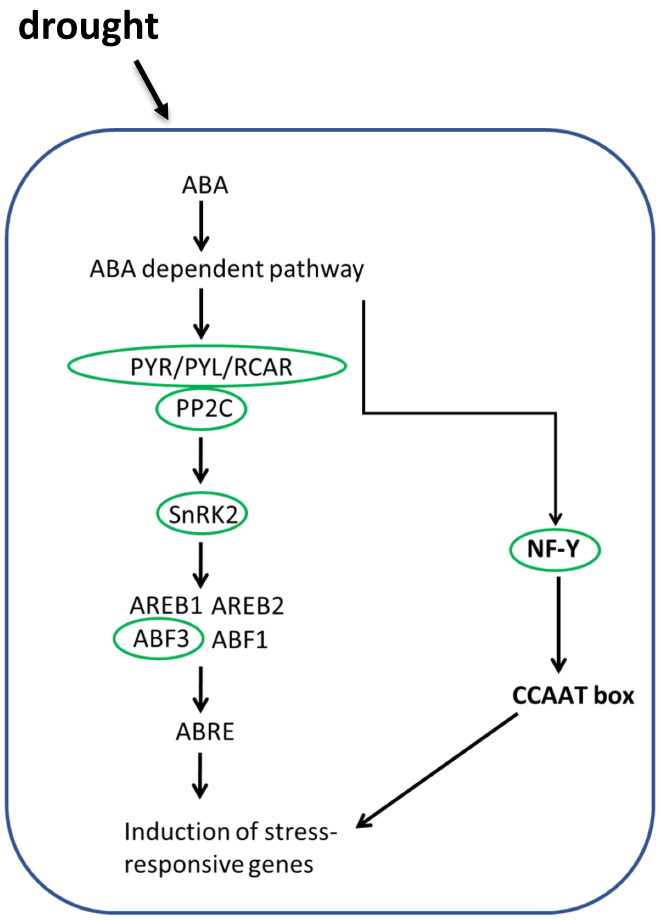

Supplementary Figure S2. SF treatment-induced ABA-dependent drought signaling in Arabidopsis. Regulation of ABA-dependent drought signaling in Arabidopsis plants primed with SF and exposed to drought stress. Under drought, ABA accumulation leads to activation of subclass III SnRK2 through the PYR/PYL/RCAR-PP2C complex. SnRK2 phosphorylates ABF3 (along with AREB1, AREB2 and ABF1) to regulate downstream genes by binding to the ABRE *cis*-element in the promotors of target genes. Green indicates upregulation in SF-primed and drought-stressed plants compared to unprimed drought-stressed plants (log2 FC). The full list of genes is given in **Table 1**.

Supplementary Figure S3

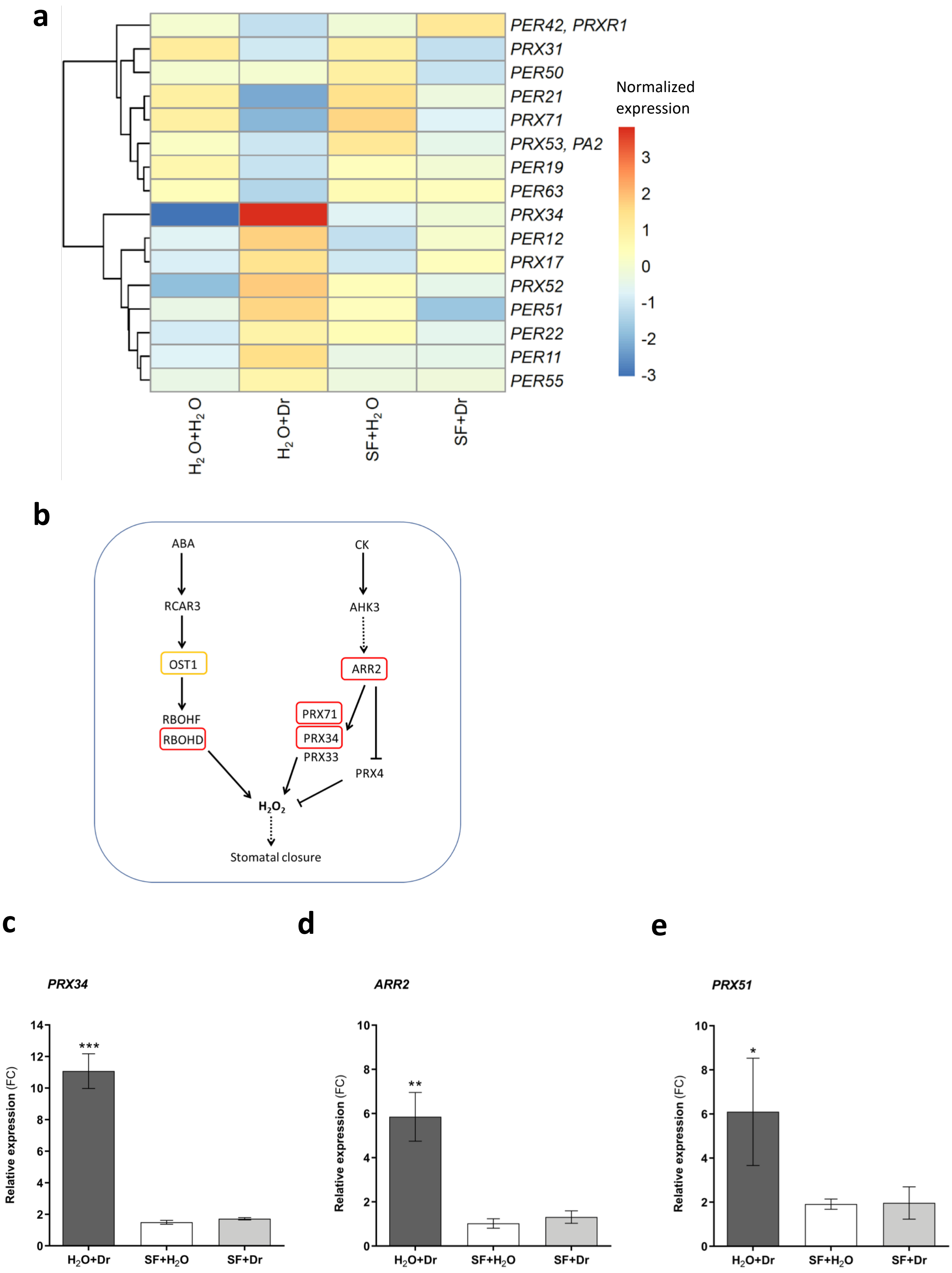

Supplementary Figure S3. Differentially expressed genes of the peroxidase family in different treatments. (**a**) Heat map of DEGs of the peroxidase family in different treatments. The color-scale denotes mean-centered normalized expression (TMM) values. (**b**) Model for the control of stomatal closure *via* accumulation of H_2O_2 in guard cells, based on results from Arnaud *et al*. (2017) and references cited in the Discussion. Under drought conditions, ABA induces stomatal closure through an accumulation of secondary messengers such as ROS in guard cells (Lim *et al*., 2015). Apart from ABA, a cytokinin-mediated signaling cascade which includes *AHK3*, *ARR2*, *PRX33*, *PRX34*, and *PRX71* activate (arrows), while *PRX4* represses the generation of ROS. As a result, H_2O_2 accumulation in guard cells leads to stomatal closure. Genes downregulated in SF-primed and drought-stressed plants at day 11 of drought are highlighted in red, whereas *OST1* (highlighted in yellow) maintained its expression at the level observed in unprimed, unstressed control plants. Dashed lines indicate indirect connections. (**c**, **d**, **e**) qRT-PCR validation of the expression of (c) *PRX34*, (d) *ARR2*, and (e) *PRX51* genes during drought (42 DAG, day 11 of drought) in Col-0 primed and unprimed plants. FC, fold change. Asterisks indicate statistically significant differences from the water-treated Col-0 control (H_2O+H_2O; one-way ANOVA, Tukey's multiple comparison test; *$p \leq 0.05$; **$p \leq 0.01$; ***$p \leq 0.001$). Error bars indicate standard error of the mean (SEM). Primer sequences used in this analysis are given in **Supplementary Table S1**. Dr, drought stress; SF, Super Fifty.

Reference:

Lim, C.W.; Baek, W.; Jung, J.; Kim, J.-H.; Lee, S.C. Function of ABA in stomatal defense against biotic and drought stresses. *Int. J. Mol. Sci*. **2015**, *16*, 15251–15270.

Supplementary Figure S4

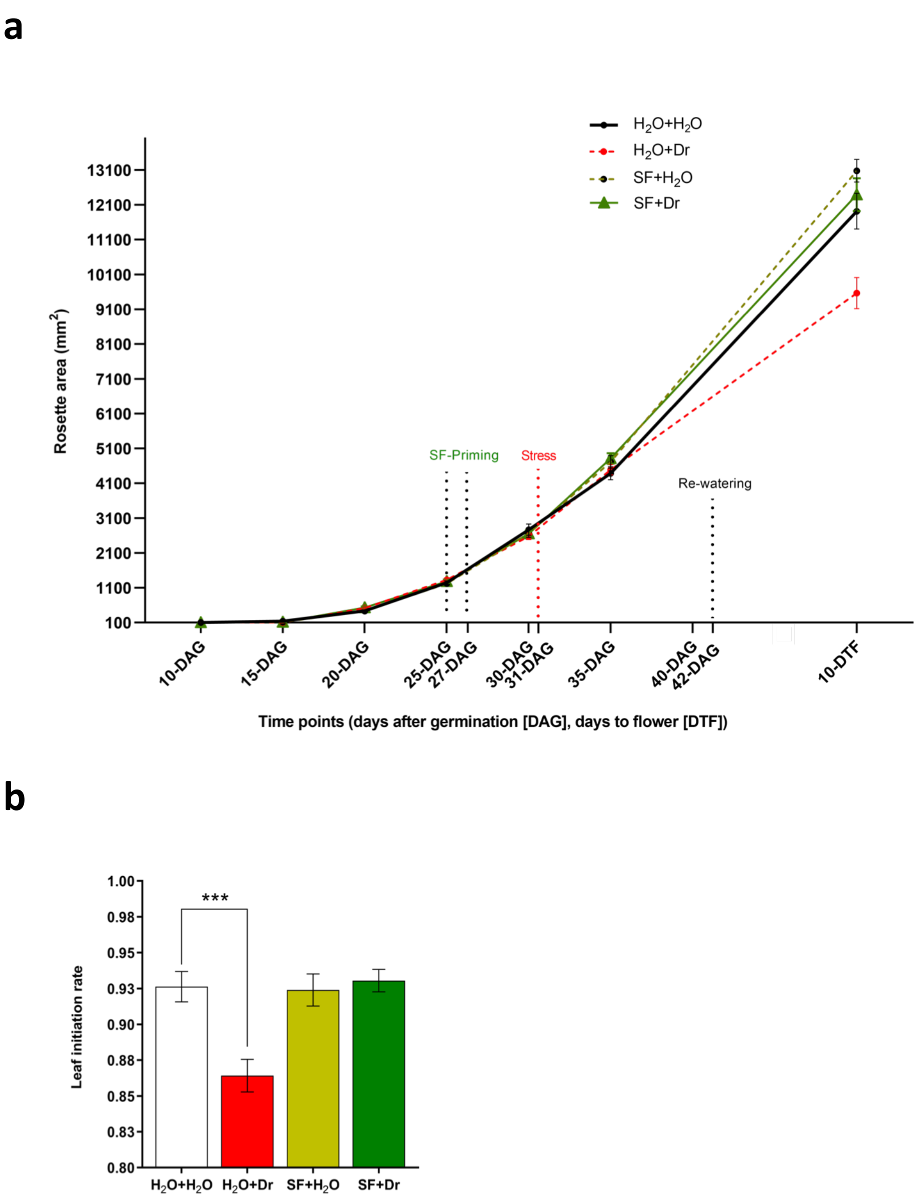

Supplementary Figure S4. Rosette growth during drought stress. (**a**) Changes in rosette area. Three-week-old Arabidopsis (Col-0) plants were primed with SF at 25 and 27 DAG, and then subjected to drought stress at 31 DAG until 42 DAG. Images of whole rosettes were taken at 5-day intervals and analyzed using ImageJ (https://imagej.nih.gov/ij). Until 30 DAG, rosette area increased similarly in SF-primed and unprimed plants. Thereafter, drought stress significantly reduced rosette growth in unprimed plants (H_2O+Dr; red interrupted line) compared to well-watered plants (H_2O+H_2O; black solid line). This reduction in rosette growth was entirely mitigated by SF priming before drought treatment. **(b)** Leaf initiation rate (LIR). Total leaf number (TLN) and days to bolting (DTB) data were used to calculate LIR. For analysis, all treatments (i.e., H_2O+Dr, SF+H_2O, SF+Dr) were compared to untreated control (H_2O+H_2O) to evaluate the effect on the leaf initiation speed. Error bars represent standard error of the mean (SEM), n = 20 (one-way ANOVA, Dunnett's multiple comparisons test). Dr, drought stress; SF, Super Fifty.

Supplementary Figure S5

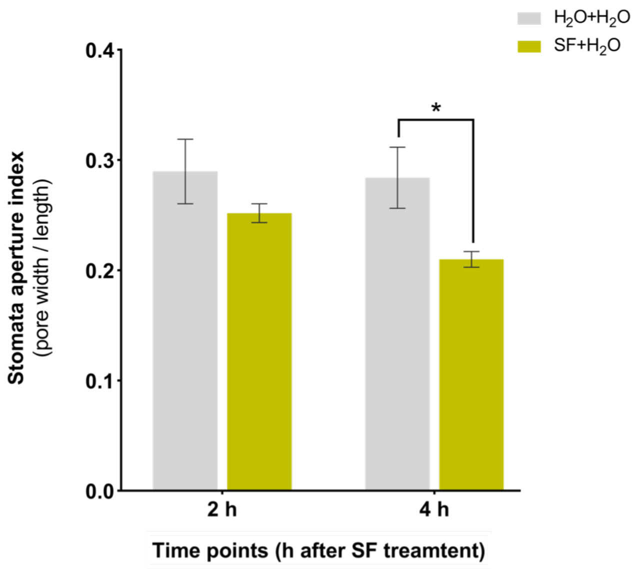

Supplementary Figure S5. SF treatment reduces stomatal aperture. Stomatal aperture in response to SF treatment. SF-treated plants showed a gradual reduction in stomatal aperture after 2 h and 4 h of foliar SF spray, compared to water-treated controls; the differences are significant after 4 h of spray. Asterisks indicate a significant difference to the corresponding water-treated leaves (*$p \leq 0.05$). Error bars show standard error of the mean (SEM); $n \geq 110$, at 2 h, and $n \geq 250$ at 4 h (Student's *t*-test with Welch's correction). SF, Super Fifty.

Supplementary Figure S6

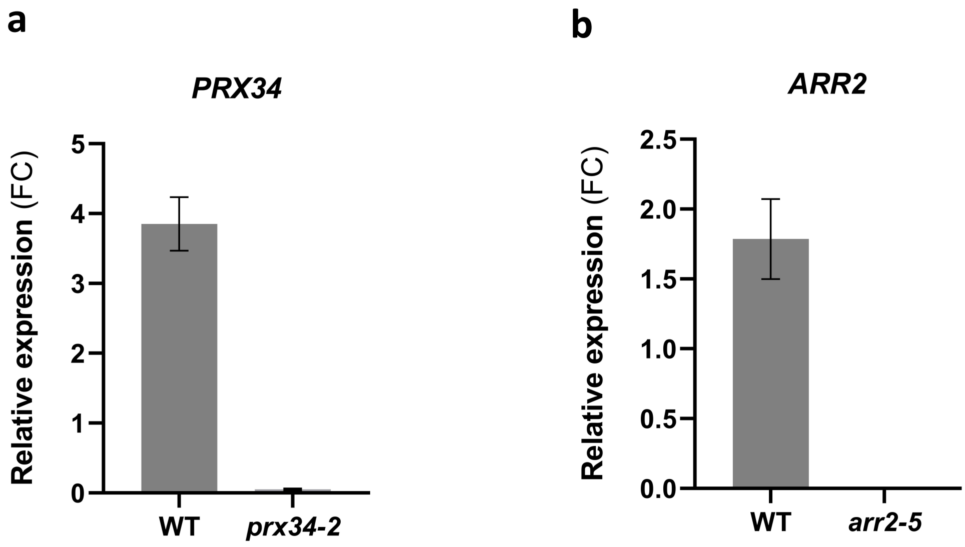

Supplementary Figure S6. Relative expression of *PRX34* and *ARR2* in knockout mutants. **(a)** Expression of *PRX34* in wild-type and *prx34-2* plants. (**b**) Expression of *ARR2* in wild-type and *arr2-5* plants. Transcript abundance was determined by qRT-PCR. *PRX34* and *ARR2* are induced by drought stress (H_2O+Dr) *vs*. well-watered conditions (H_2O+H_2O) in wild-type plants but not their respective mutants. Error bars indicate standard error of the mean (SEM). FC, fold change.

Supplementary Figure S7

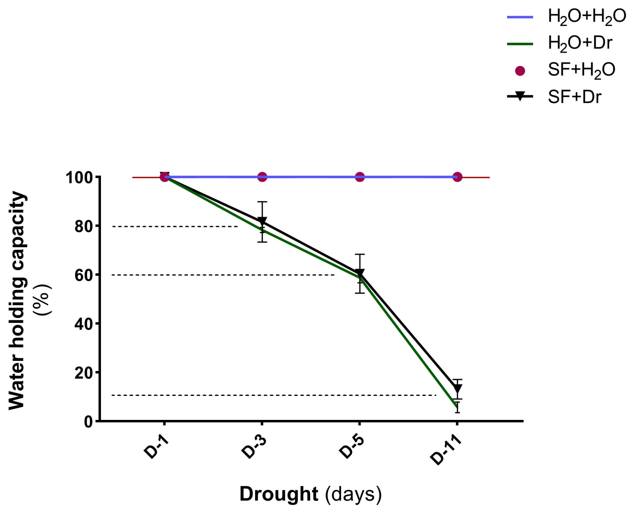

Supplementary Figure S7. Water holding capacity. Soil moisture contents were determined and calculated as % water holding capacity in SF-primed (SF+H_2O, SF+Dr) and unprimed (H_2O+H_2O, H_2O+Dr) plants. Dr, drought stress; SF, Super Fifty.
