# Supplementary material for: Priming with a Seaweed Extract Strongly Improves Drought Tolerance in Arabidopsis
Source: Int J Mol Sci. 2021 Feb 2;22(3):1469. doi: 10.3390/ijms22031469 (PMC7867171; doi:10.3390/ijms22031469)
Supplement: Supplementary file 1 [file ijms-22-01469-s001.zip › Suppl files/Supplementary Table S1 final.docx]

**Supplementary Table S1.** **Oligonucleotide used in this study.**

| **Primers for qRT-PCR** | | |
| --- | --- | --- |
| **No.** | **Name** | **Oligonucleotide sequence** (5’ - 3’) |
| 1 | *PRX34*-1*-*F | ATCGTCCTTCTGATCTCGTTGCT |
| 2 | *PRX34*-1*-*R | GATCAAAATCTACCAAGGCACTTC |
| 3 | *PRX34*-2-F | TCACCCCTACCTTCTACGATAG |
| 4 | *ARR2*-1-F | CCTTCTCTGATCGTTCGTTTTCTG |
| 5 | *ARR2*-1-R | ATCAACGACAAGAACTCGAAGATTC |
| 6 | *ARR2-*F | GAATCAGCTAGGCGTTGACAAAG |
| 7 | *ARR2-*R | GCAAGGCTCTGAGGAGGGAG |
| 8 | *PRX51-*F | ATTCTCTCTCTTTTTCTCGCCATCA |
| 9 | *PRX51-*R | AAGTGCATTGAGTTGGTTGAGATCA |
| **Primers for mutant lines** | | |
| 10 | *prx34*-a-F | TCCGTTAGCGTTCCACATTAGTTGT |
| 11 | *prx34*-a-R | TTGAGCAGCGGACAAAGATGCATG |
| 12 | *prx34*-b-F | CACCCCTACCTTCTACGATAG |
| 13 | *prx34*-b-R | CCATTTGTTCCTCTGAAGCAAG |
| 14 | *prx34-*c-F | GACATCCGAAAAATACATCCC |
| 15 | *prx34-*c-R | CCATACCGAAATAATATTTGTATACCG |
| 16 | *arr2-5*-F | GCCATGCCCTTCATAAATCTTC |
| 17 | *arr2-5*-R | ACAATGCGCTCTCTGCTCTGT |
| 18 | *arr2-5*-LP-F | GTTTCAAGCTCCTTGAACACG |
| 19 | *arr2-5*-RP-R | GCTGCTCTGAACATTCTGTCC |
| 20 | GABI_8474-T-DNA | ATAATAACGCTGCGGACATCTACATTTT |
| **Primers for RNA *in situ* hybridization** | | |
| 21 | *HISTONE4*-F | ATGCAGGAAGAGGAAAAGGAGG |
| 22 | *HISTONE4*-R | TCAACCACCAAATCCATATAGAG |
| 23 | *RD26*-F | ATGGGTGTTAGAGAGAAAGAT |
| 24 | *RD26*-R | TCATTGCCTAAACTCGAATG |
